# Author Correction: In situ ultrastructures of two evolutionarily distant apicomplexan rhoptry secretion systems

**DOI:** 10.1038/s41467-021-26483-6

**Published:** 2021-10-21

**Authors:** Shrawan Kumar Mageswaran, Amandine Guérin, Liam M. Theveny, William David Chen, Matthew Martinez, Maryse Lebrun, Boris Striepen, Yi-Wei Chang

**Affiliations:** 1 Department of Biochemistry and Biophysics, Perelman School of Medicine, University of Pennsylvania, Philadelphia, PA USA; 2 Department of Pathobiology, School of Veterinary Medicine, University of Pennsylvania, Philadelphia, PA USA; 3 LPHI, UMR 5235 CNRS, Université de Montpellier, Montpellier, France

**Keywords:** Biophysics, Parasite biology, Cryoelectron tomography

Correction to: *Nature Communications* 10.1038/s41467-021-25309-9, published online 17 August 2021.

Supplementary Movies 1–5 and Description of Additional Supplementary Files were missing from this article and have now been uploaded. The original article has been corrected.

## Supplementary information

Supplementary Movie 1

Supplementary Movie 2

Supplementary Movie 3

Supplementary Movie 4

Supplementary Movie 5

Description of Additional Supplementary Files

