# Supplementary material for: In situ ultrastructures of two evolutionarily distant apicomplexan rhoptry secretion systems
Source: Nat Commun. 2021 Aug 17;12:4983. doi: 10.1038/s41467-021-25309-9 (PMC8371170; doi:10.1038/s41467-021-25309-9)
Supplement: Supplementary file 1 — Supplementary Information [file 41467_2021_25309_MOESM1_ESM.pdf]

SUPPLEMENTARY INFORMATION

Supplementary information guide

1. Supplementary Discussion: A membrane fusion model for apicomplexan rhoptry secretion.
2. Supplementary Figures
    - Supplementary Fig. 1: Organization of rhoptry in *C. parvum.*
    - Supplementary Fig. 2: Organization of rhoptry in *T. gondii.*
    - Supplementary Fig. 3: Microneme filaments in *C. parvum*.
    - Supplementary Fig. 4: Additional examples for anterior filaments of *T. gondii* rhoptries.
    - Supplementary Fig. 5: Subtomogram averaging scheme for anterior filaments of *T. gondii* rhoptries and intermediate results.
    - Supplementary Fig. 6: Associations between the rhoptry, the apical vesicle (AV), and the plasma membrane.
    - Supplementary Fig. 7: Microtubule-associated vesicles (MVs) are possibly the precursors of the apical vesicle (AV) in *T. gondii*.
    - Supplementary Fig. 8: Organization of intraconoidal microtubules (IMTs) and associated structures in *T. gondii*.
    - Supplementary Fig. 9: Examples for positioning of microtubule-associated vesicles (MVs) on intraconoidal microtubules (IMTs).
    - Supplementary Fig. 10: Apical vesicle (AV) is consistently organized in front of the intraconoidal microtubules (IMTs).
    - Supplementary Fig. 11: Rhoptry secretory apparatus (RSA) is important for proper anchoring of apical vesicle (AV) to plasma membrane to ensure rhoptry secretion.
    - Supplementary Fig. 12: Subtomogram averages of the rhoptry secretory apparatus (RSA) in *C. parvum* in differently treated conditions.
    - Supplementary Fig. 13: Subtomogram averaging schemes for the rhoptry secretory apparatus (RSA) of *C. parvum* and *T. gondii.*
    - Supplementary Fig. 14: Ultrastructures of rhoptry secretory apparatus (RSA) in *C. parvum* and *T. gondii* – additional interpretations.
    - Supplementary Fig. 15: Relationship between conoid extension and the ultrastructure of rhoptry secretory apparatus (RSA).
3. Supplementary Tables
    - Supplementary Table 1: Measurements of diameter from individual rhoptry necks in *C. parvum.*
4. Supplementary References

1. Supplementary Discussion

A membrane fusion model for apicomplexan rhoptry secretion.

Surface rosette structure has been previously shown to be involved in the exocytosis of extrusomes in free-living ciliates such as trichocysts in *Paramecium spp*. and mucocysts in *Tetrahymena spp*.[1-12]. The process involves the fusion of the plasma membrane and the opposing extrusome membrane, possibly with the help of uncharacterized densities (termed as connecting material) that were occasionally seen sandwiched between the two opposing membranes[1]. This arrangement, together with the 8-fold rotational symmetry of the rosette, resembles that of the apicomplexan RSAs found in this study bridging the plasma membrane and AV membrane. Since ciliates and apicomplexans belong to the same Alveolata infrakingdom and removing (or mutating) the conserved Nd9 protein in both *Paramecium*[2] and *T. gondii*[13] showed disruption of the rosette and secretion, a membrane fusion mechanism is likely shared among these alveolates. In the case of apicomplexans, the membrane fusion would occur between the AV and the plasma membrane. These findings support the idea of a common ancestral eukaryotic secretion machine that has evolved as per the needs of the extant unicellular eukaryotes. One of the readily noticeable adaptations in apicomplexan rhoptry secretion is the presence of the AV (absent in ciliate extrusomes). The AV could provide an additional layer of control in rhoptry secretion (i.e., rhoptry docking and fusion to the AV in addition to RSA-mediated membrane fusion of the AV to the plasma membrane) and coordinate secretion from more than one rhoptry (e.g., in *T. gondii*). Freeze fracture studies of ciliates have suggested disassembly of the rosette during exocytosis[1,3,5,7]. By analogy, for rhoptries in apicomplexans to secrete, one mechanistic hypothesis could be that the anchors in the RSA clamping and holding the AV away from the plasma membrane disassemble in order for membrane fusion to proceed. Alternatively, radial dispersion or torqueing motion of the anchor-I in the RSA could exaggerate the twist in the anchors-II and -III, thereby pulling the AV closer to the plasma membrane for fusion. These working models for RSA-mediated membrane fusion remain to be validated.

The presence of an elaborate RSA is supportive of the tight regulation of rhoptry secretion, which happens during the initial phase of host invasion following microneme secretion and likely requires host cell attachment (shown in *T. gondii*)[14-16]. Thus, the signal for rhoptry secretion most likely comes from the cell exterior. It is necessary for this signal to be received, transmitted across the plasma membrane and translated into a mechanical force to bring opposing membranes in close proximity for fusion. The RSA, with its tightly interacting extracellular, transmembrane, and intracellular components, is well equipped to perform such a function. It is worth noting that the rhoptry proteins are not merely secreted outside the parasite but are delivered into the host. In fact, a widely accepted model proposes a direct delivery of rhoptry proteins into the host cytoplasm[14,17]. To achieve that, it is possible that the posterior central channel (in *C. parvum*) and the anterior central channel (in both *C. parvum* and *T. gondii*) could serve to breach the host plasma membrane by inserting themselves into the latter and providing a transmembrane route. Consistent with this model, a ~40 nm pore was seen in the host plasma membrane during *T. gondii* invasion[16,18]. Also consistent with this model is that the AV (that organizes the potential pore-forming anterior and posterior central channels) seems to be unique to apicomplexan rhoptries that secrete proteins into host cells and absent in extrusomes of free-living ciliates that secrete proteins just outside their cell.

It is possible that the rosette's central density, which contains a putative pore could help with ion influx across the plasma membrane for signaling. Ca^{2+} is of particular interest since the related rosettes in *Paramecium* are implicated in Ca^{2+} influx[4,12,19,20] and there are Ca^{2+} binding proteins important for rhoptry secretion, showing proximal localization to the RSA in *T. gondii* (i.e., Ndp2[13] and Fer2[21]). However, Ca^{2+}-mediated regulation of membrane fusion might be different between the two systems. While Ca^{2+} influx is concomitant with a massive rearrangement of rosettes and exocytosis in Ciliata[3,5-7,12], the two RSAs we analyzed did not show discernable changes in response to Ca^{2+} or in the context of other Ca^{2+}-dependent activity – we found that the Ca^{2+} ionophore A23187 induces no large-scale changes in the RSA ultrastructure (or the rhoptry system as a whole) in *C. parvum* (Supplementary Fig. 12e, f; Methods); and the extension of the conoid, which is a Ca^{2+} dependent process[22], seems to occur

without affecting the RSA ultrastructure and the anchoring of AV on the plasma membrane in *T. gondii* (Supplementary Fig. 15). Consistent with our results, a spike in Ca^{2+} level alone is known to be insufficient to trigger rhoptry secretion without host cell attachment[16].

2. Supplementary Figures

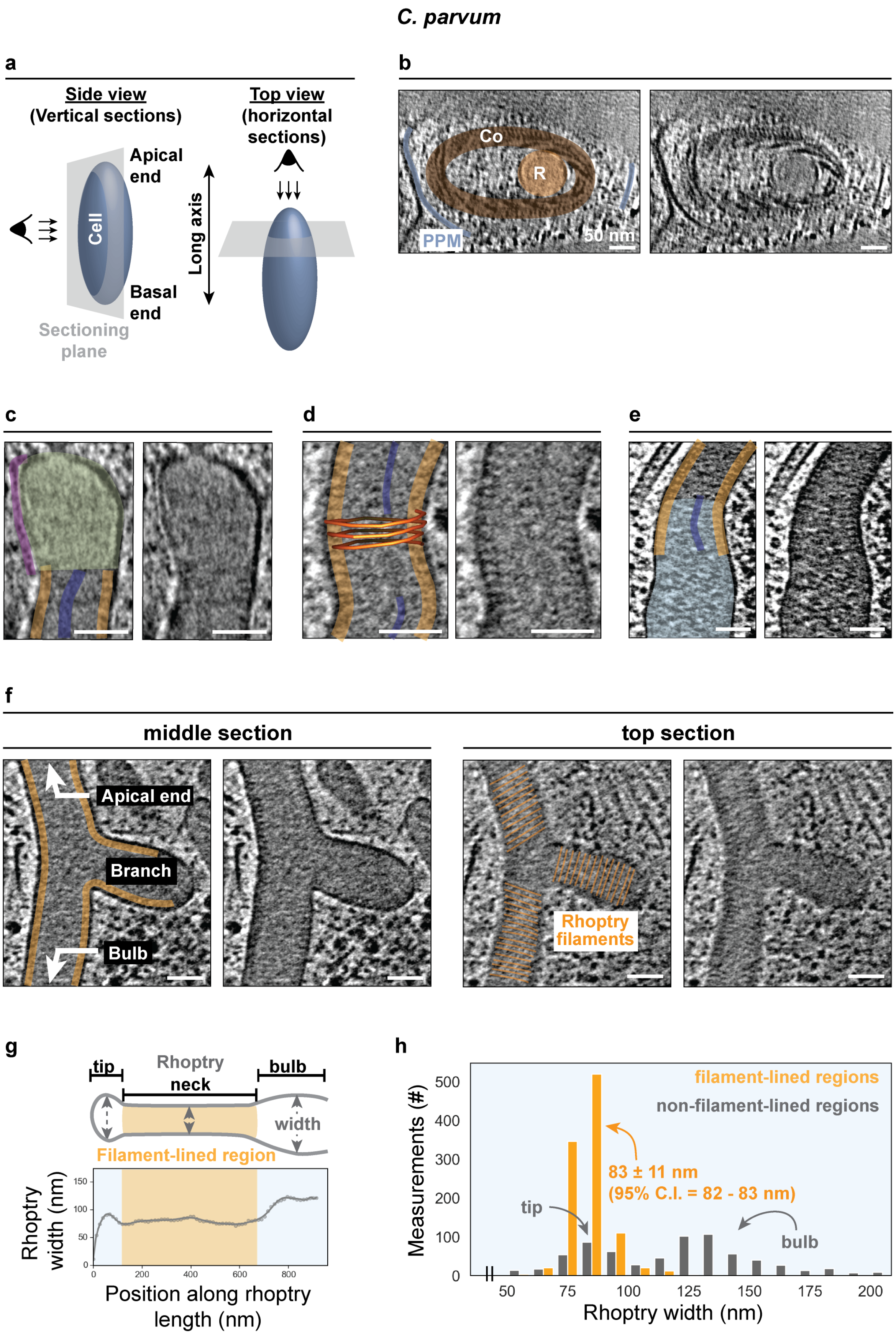

Supplementary Fig. 1: Organization of rhoptry in *C. parvum.*

(a) A schematic showing the orientation of the computational sectioning planes with respect to the parasite body to reveal side views and top views. (b) Top view of the arrangement of rhoptry (R; orange) with respect to the conoid (Co; brown). Parasite plasma membrane (PPM) is shown in light blue in (b). (c-e) Rhoptry organization in the tip (c), the neck (d) and the bulb (e) showing features such as tip densities (purple), helical neck filaments (orange; one-start helical filament in (d)), and dense luminal strands (dark blue). During cryo-ET imaging the bulb usually shows the first signs of radiation damage (light blue in (e)) of all the cellular compartments suggesting a dense packing of proteins, although rhoptry necks and micronemes also subsequently show such damage. Similar damage can also be noted in the bulb region in Fig. 1b. (f) Organization of filaments at the branch points in the rhoptry neck. (g) Width measurements along the length of a representative rhoptry (a total of 26 rhoptries in as many cells analyzed) with an overlay of filament-lined region (orange). The filament-lined region correlates well with the region of the rhoptry with a uniform width (the neck). (h) A histogram of width measurements made every ~10 nm along the filament-lined and non-filament-lined regions from all 26 rhoptries showing a tight correlation between presence of the filament and a rhoptry diameter of 83 ± 11 nm (mean ± std; with a 95% confidence interval or C.I. of 82-83 nm). Scale bars in all panels are 50 nm.

T. gondii

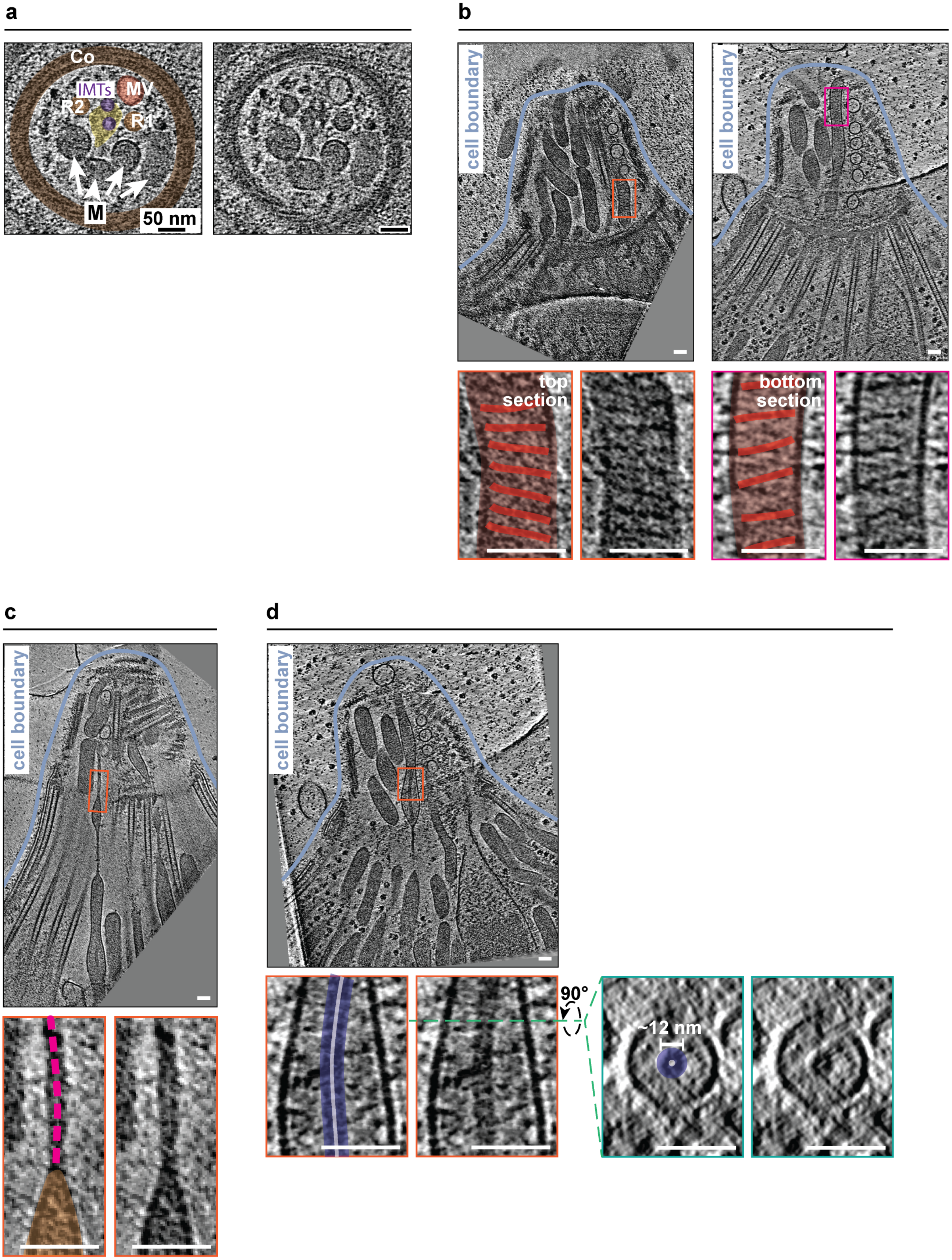
a
Co
IMTs
MV
R2
R1
M
50 nm
b
cell boundary
cell boundary
top section
bottom section
c
cell boundary
d
cell boundary
90°
~12 nm

T. gondii

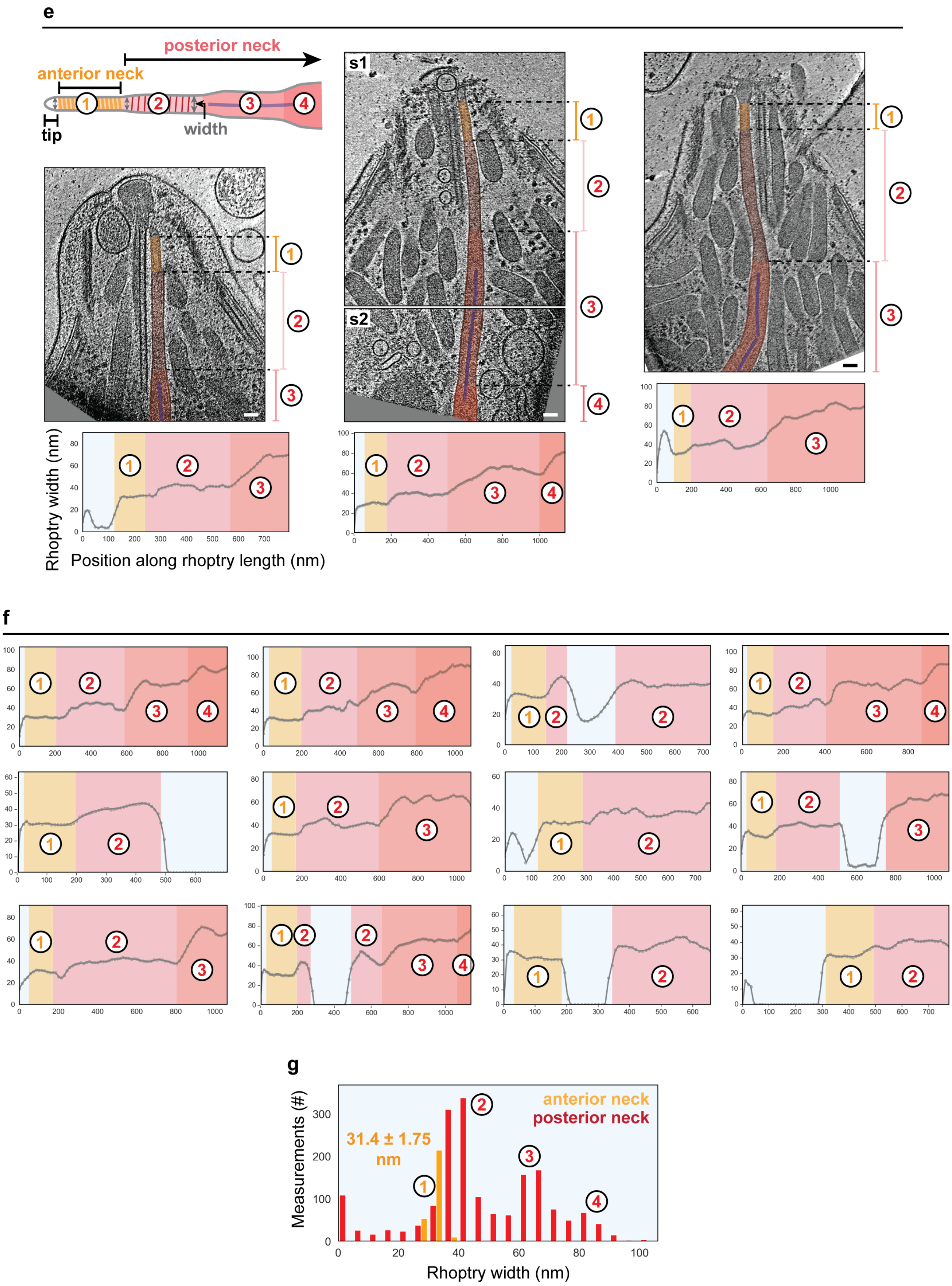
e
posterior neck
anterior neck
tip
width
s1
s2
Rhoptry width (nm)
Position along rhoptry length (nm)
f
g
anterior neck
posterior neck
31.4 ± 1.75 nm
Measurements (#)
Rhoptry width (nm)

Supplementary Fig. 2: Organization of rhoptry in *T. gondii.*

(a) Top view of the arrangement of two rhoptries (R_1 and R_2; orange) with respect to the apical complex including the conoid (Co; brown), micronemes (M), intraconoidal microtubules (IMTs; purple), microtubule-associated vesicle (MV; red), and fibrous densities around the IMTs (yellow). (b-d) Representative cells (n > 25 cells) and enlarged views of the feature of interest (boxed region in orange) are shown. (b) Posterior neck of rhoptries showing left-handed helical filaments of variable geometries (red). (c) A local constriction (pink dashed line) along the length of a rhoptry showing the two opposing membranes in cross-section collapsed together. (d) A dense luminal strand (dark blue) showing a possible hollow center (better visible in the cross-sectional top view on the right). (e) Width measurements along the length of 3 representative rhoptries (a total of 31 rhoptries were analyzed from 19 cells). Orange indicates the anterior neck (marked by zone 1) and shades of red indicate the posterior neck (marked by zones 2, 3 and 4). The rhoptries show a stepwise increase in width (zone 1 < zone 2 < zone 3 < zone 4) as it approaches the bulb (outside the field of view for width measurement). Correspondingly, these regions display different ultrastructural features (zone 1 – anterior filament, zone 2 – posterior filament, zone 3 – dense strands). Rhoptry width measurements close to 0 indicate constrictions. s1 and s2 indicate two different sections from the same tomogram for the middle cell. (f) More examples of rhoptry width measurements. (g) A histogram of width measurements of the anterior and posterior neck regions from all 31 rhoptries. Presence of the anterior filament tightly correlates with a rhoptry diameter 31.4 ± 1.75 nm (mean ± std). The multi-modal distribution of widths for the posterior neck corresponds to zones 2-4, suggesting the presence of different membrane remodeling elements along the length of the rhoptries (possibly different helical filaments). Source data for this figure are provided as a Source Data file. Additionally, three-dimensional (3-D) volumes for raw tomograms of (a-e) panels are included in the repository referenced under “Data availability”. Scale bars in all panels are 50 nm.

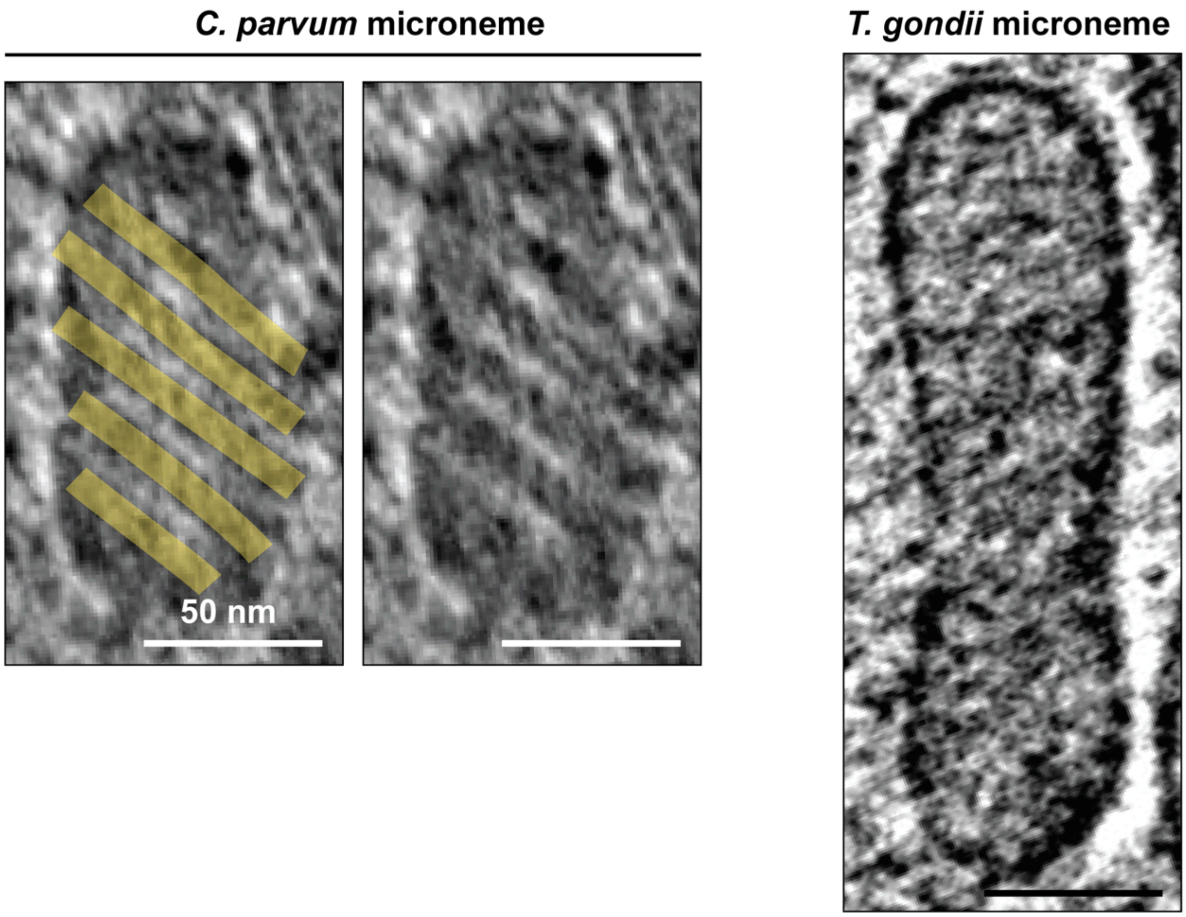

Supplementary Fig. 3: Microneme filaments in *C. parvum*.

Micronemes of *C. parvum* show multi-start helical filaments (yellow) underneath their bounding membrane. These have been previously described as quasi-helical structures[23]. They are likely to contribute to the shape of micronemes but their actual function is unknown. Their *T. gondii* counterparts do not exhibit such clear filaments. Scale bars in all panels are 50 nm.

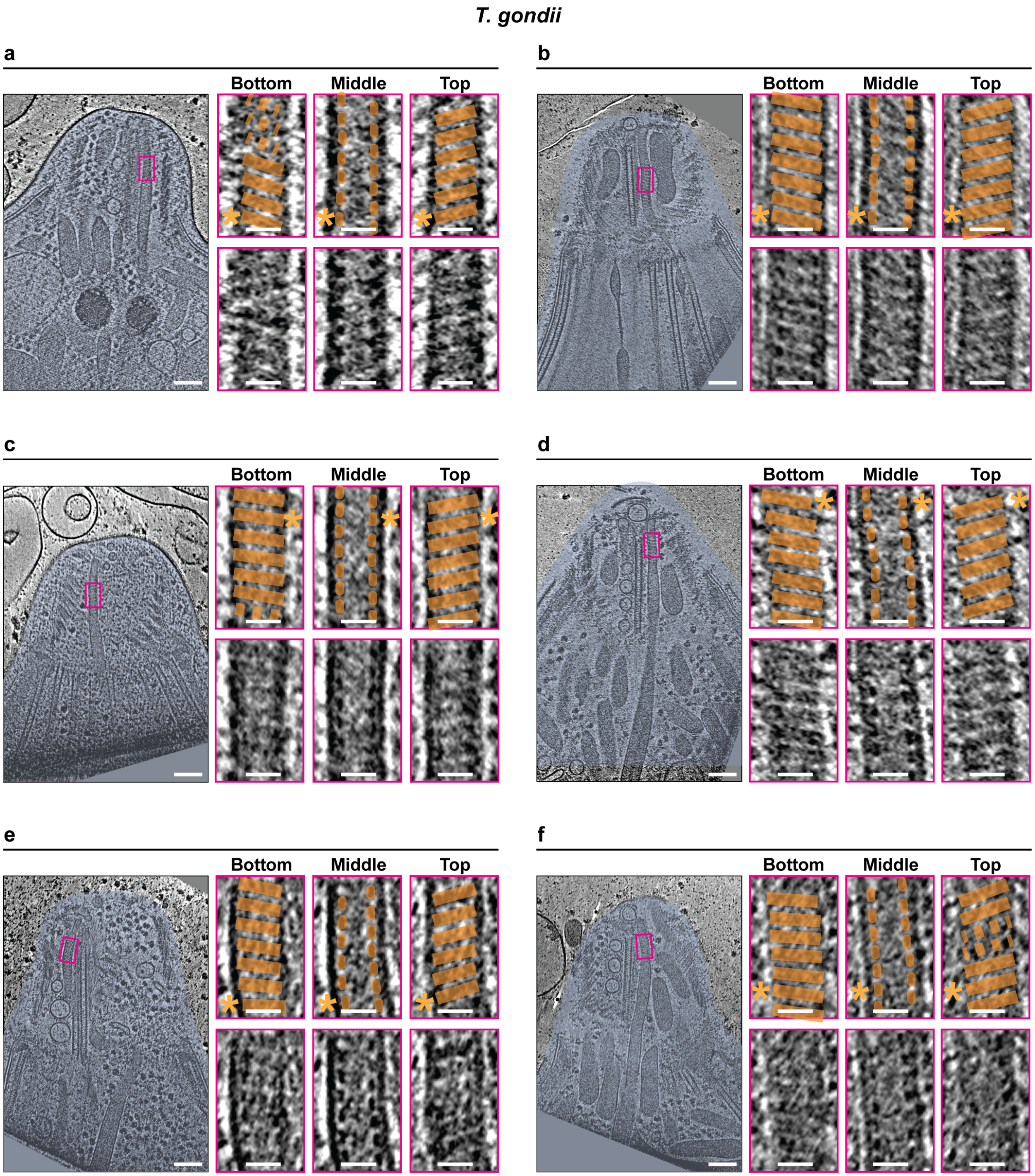

Supplementary Fig. 4: Additional examples for anterior filaments of *T. gondii* rhoptries.

(a-f) These six representative cells, chosen from > 25 tomograms with good signal-to-noise, consistently show helical geometry for anterior rhoptry filaments in *T. gondii*. For each example, enlarged views of the filaments (smaller panels on the right) come from the boxed region (in pink) in the corresponding cell (larger panels on the left). The cell interior is shaded blue to help differentiate from the exterior. Side view tomogram sections (top, middle and bottom) through the anterior rhoptry neck

regions show the expected arrangement for a helical filament (orange overlays; ambiguous arrangements are shown as orange dashed stripes). Orange asterisks point to the same region of the filament at one of the helical turns in each example. Source data for this figure are provided as a Source Data file. Scale bars in all the larger and smaller panels are 100 nm and 20 nm, respectively.

T. gondii anterior filament subtomogram averaging scheme

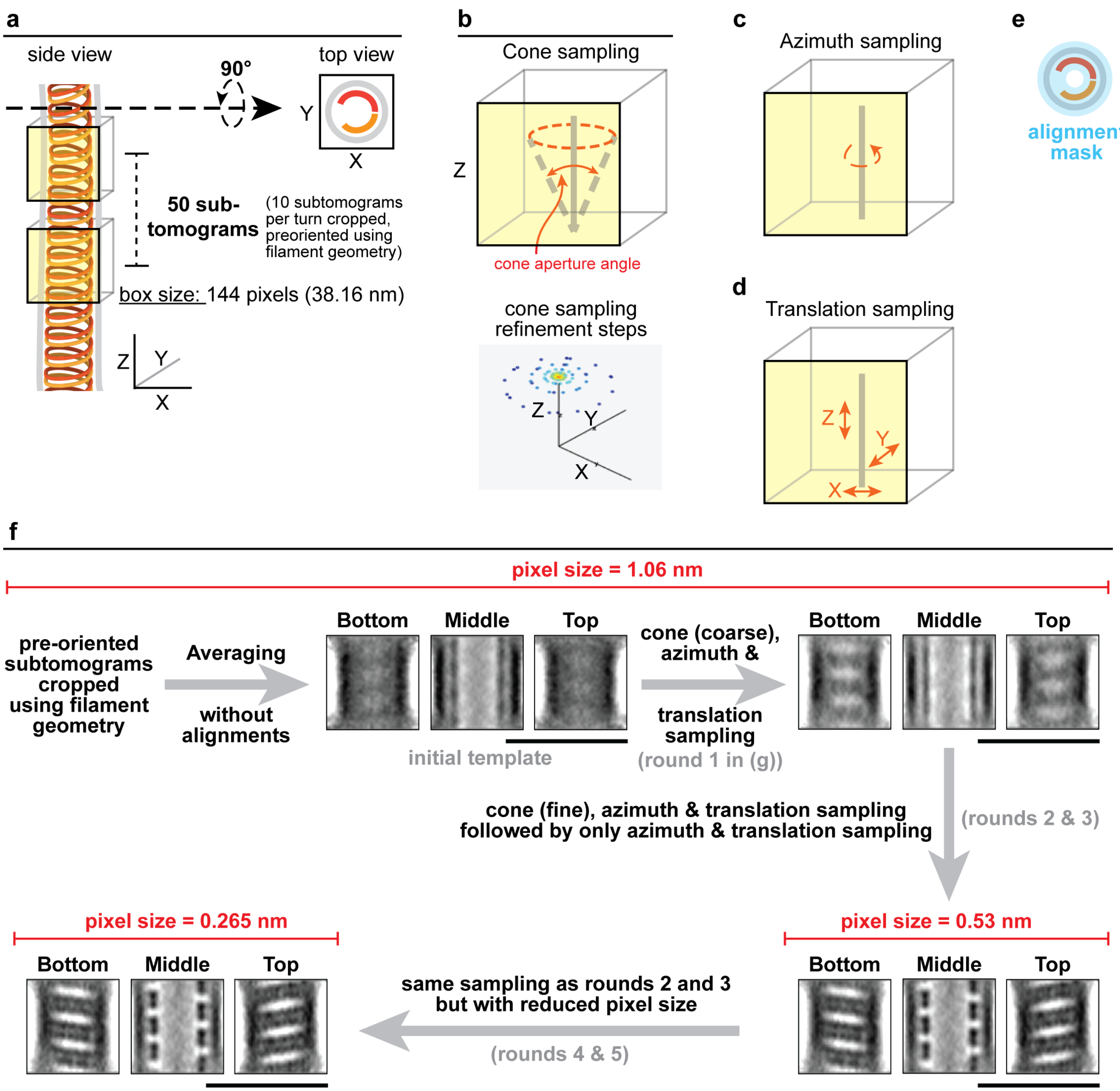

g

	round 1	round 2	round 3	round 4	round 5
iterations	1	1	1	1	2
references	1	1	1	1	1
cone aperture	30	3	0	3	0
cone sampling	10	1	1	1	1
azymuth rotation range	36	36	36	36	36
azymuth rotation sampling	12	12	12	12	12
refine	7	7	7	7	7
refine factor	2	2	2	2	2
high pass	2	2	2	2	2
low	48	32	32	32	32
symmetry	c1	c1	c1	c1	c1
Particle dimensions.	36	72	72	144	144
* shift limits	15 1...	15 1...	15 1...	15 1...	15 1...
shift limiting way	2	2	2	2	0
separation in tomogram	0	0	0	0	0
basic MRA	0	0	0	0	0
threshold parameter	0.66	0.66	0.66	0.66	1
treshold modus	5	5	5	5	5

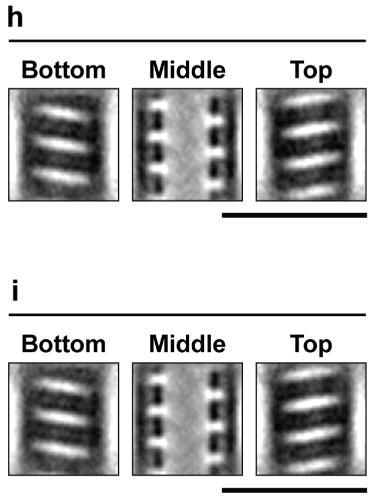

Supplementary Fig. 5: Subtomogram averaging scheme for anterior filaments of *T. gondii* rhoptries and intermediate results.

(a) A schematic for subtomogram extraction while generating an average for the anterior rhoptry filament. In short, cubical boxes were used to pick short regions of the filament (comprising of ~4 helical turns) by sliding it along the rhoptry neck. 10 subtomograms were chosen for every helical turn, 1.1 nm away from each other along the long axis of the rhoptry (filaments showed a helical pitch of ~11 nm). To roughly orient these subtomograms (i.e., to synchronize the starting phases of their helices before any alignment procedures were performed) adjacent subtomograms were rotated by 36° about the rhoptry long axis with respect to each other. (b, c) Types of angular and (d) translational search during alignment of subtomograms using the software, Dynamo. Every type of search is refined over several iterations by progressively constraining the search space as illustrated for cone sampling in lower panel of (b). (e) Alignment mask – a hollow cylinder (in cyan) that included the rhoptry membrane and membrane associated filament densities. (f) Steps for alignment of subtomograms and intermediate results – First, pre-oriented subtomograms were directly averaged without alignments to generate an initial template. Second, coarse cone sampling along with fine azimuth and translational search on four-times binned subtomograms with a pixel size of 1.06 nm. Third, fine cone sampling along with fine azimuth and translational search on twice-binned subtomograms (pixel size of 0.53 nm). Fourth, repeating step 3 using unbinned subtomograms (with original pixel size of 0.265 nm). Each of these steps included 7 iterations for refining search parameters by 2 while using the best two-thirds of the aligned subtomograms (based on cross-correlation scores) were used to generate a template for the subsequent iteration. (g) Dynamo table summarizing the alignment parameters for all the steps detailed above – step 1: no alignments; step 2: round 1; step 3: rounds 2 and 3; and step 4: rounds 4 and 5. *Shift limits are 15 in all three axes for all 5 rounds. (h) A second average independently generated for the same filament (using the same procedure and 1,100 subtomograms from a different set of 7 tomograms) shows a similar result to the first average (compare with panel (f) or Fig. 1h), validating our observations. (i) Combining subtomograms from both averages did not improve the details any further.

3-D volumes for all averages and intermediate results are included in the online repository referenced under “Data availability”. All scale bars are 50 nm.

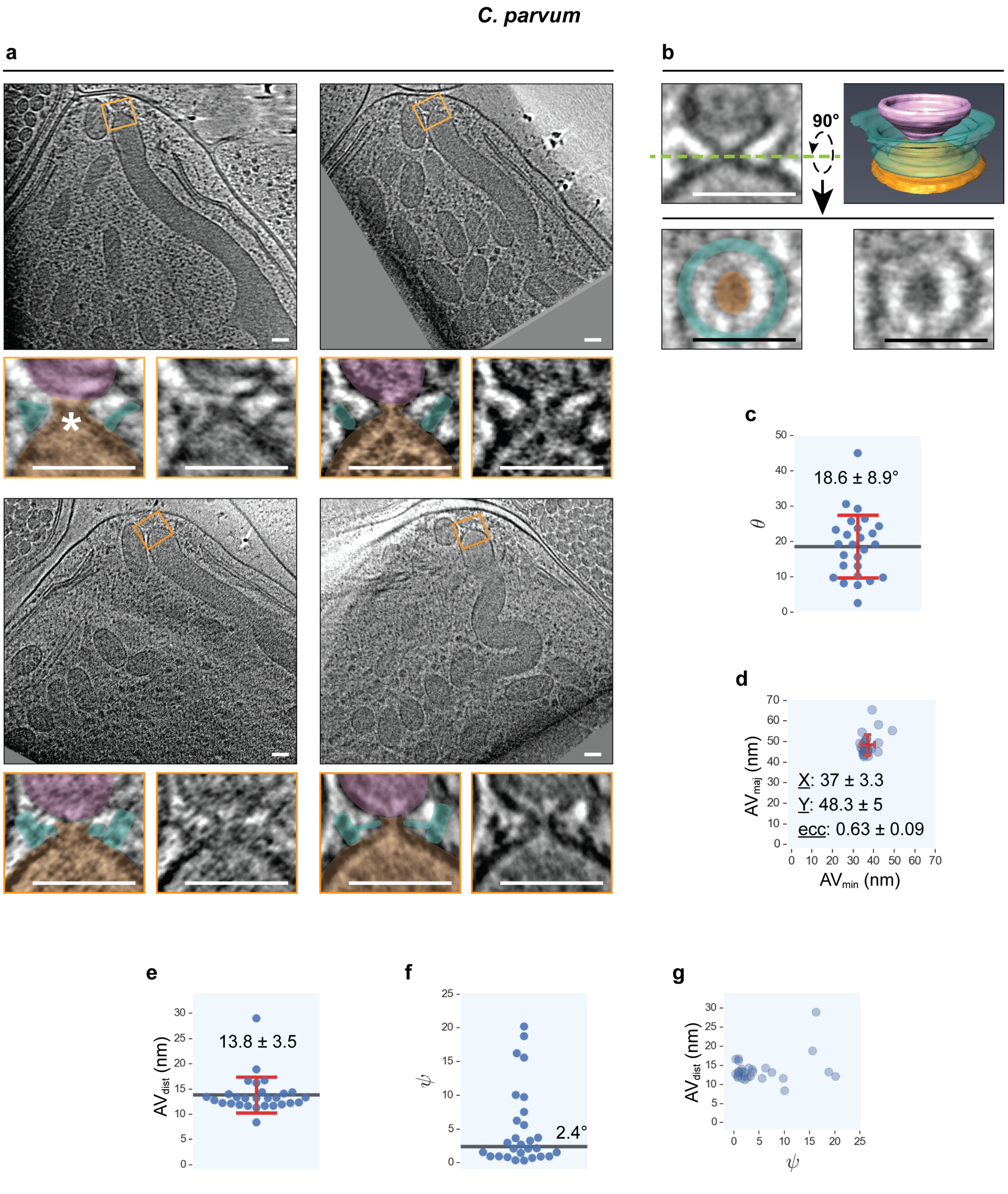
C. parvum
a
b
90°
c
18.6 ± 8.9°
θ
d
X: 37 ± 3.3
Y: 48.3 ± 5
ecc: 0.63 ± 0.09
AV_{maj} (nm)
AV_{min} (nm)
e
13.8 ± 3.5
AV_{dist} (nm)
f
ψ
2.4°
g
AV_{dist} (nm)
ψ

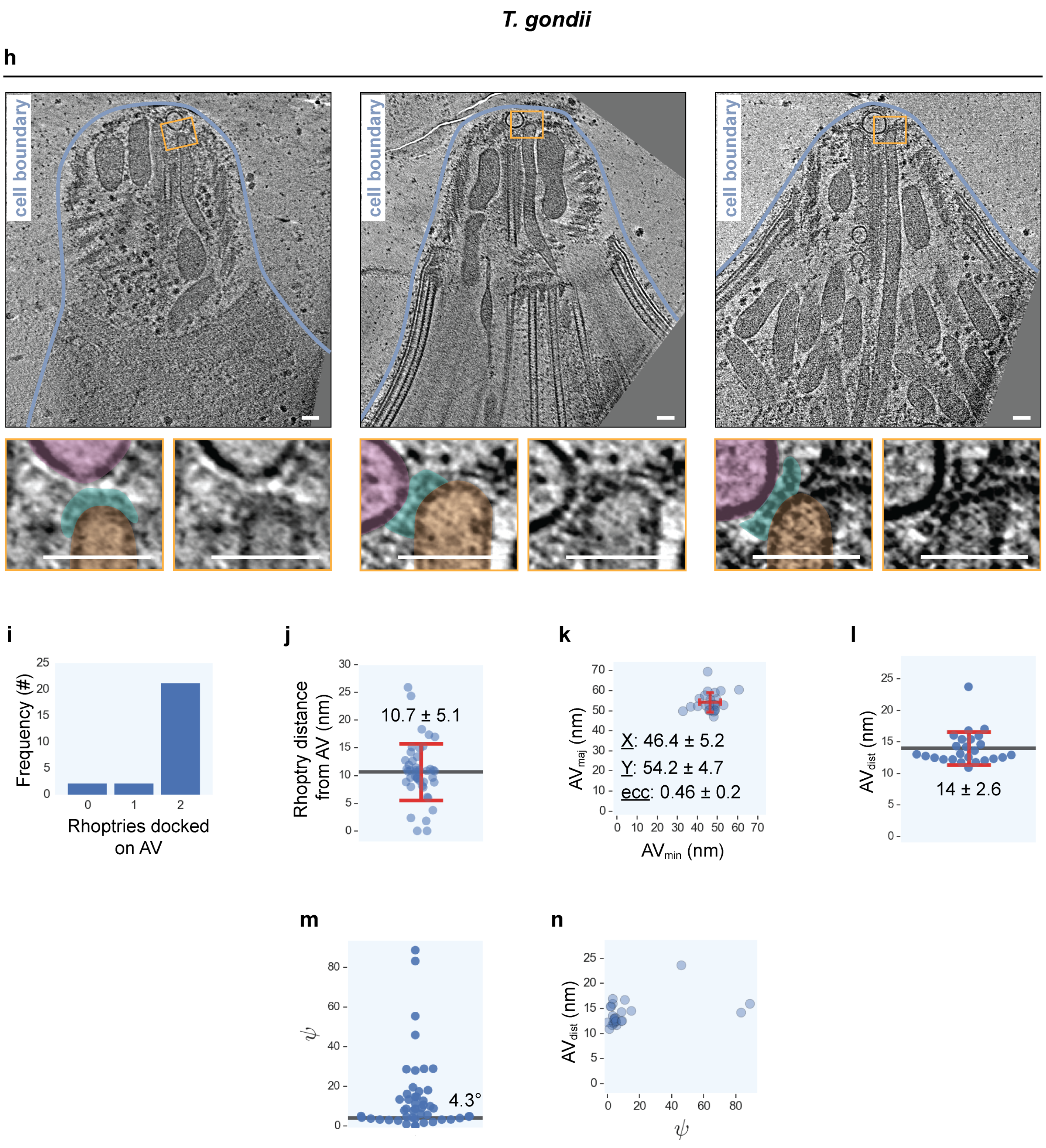

Supplementary Fig. 6: Associations between the rhoptry, the apical vesicle (AV), and the plasma membrane.

Representative images and analyses related to the apical vesicle (AV) for (a-g) *C. parvum* and (h-n) *T. gondii*. In both organisms AV was observed to associate with rhoptry tips and plasma membrane; > 150 cells of *C. parvum* and > 100 cells of *T. gondii* were qualitatively assessed. (a, h) Enlarged views of

rhoptry docking/fusion with the AV (from orange boxed regions of representative cells) are displayed. Sample sizes for quantifications: (c-g) n = 28 cells for *C. parvum* and (i-n) 25 cells for *T. gondii*, respectively. (a) In *C. parvum*, rhoptry (orange) fuses with the AV (pink) to form the AV neck (in 27 of 28 observed cells; * denotes blocked passage in some cells) surrounded by a "collar"-like density (cyan), while (h) in *T. gondii*, the rhoptries simply dock on to the AV via tip densities (cyan). More often, 2 rhoptries are simultaneously docked on to the AV in *T. gondii* (i; in 21 out of 25 cells) whose docking distances are plotted in (j); rhoptries farther than 50 nm were considered not docked and not plotted). (b) Subtomogram average of the AV neck enhanced the contrast of the collar (upper left: side view; lower row: top view; upper right: 3-D segmented volume). However, the averaging resulted in the loss of some fine features of the collar and the neck passage seen in the individual examples in (a), suggesting conformational variation. (c) Orientation of the AV neck with respect to the minor axis of AV. (d, k) Length measurements of the AV major axis (AV_{maj}) and minor axis (AV_{min}). (e, l) AV's anchoring distance from the plasma membrane (AV_{dist}). (f, m) AV's orientation with respect to the plasma membrane; the major axis of AV in *T. gondii* and the minor axis of AV in *C. parvum* were used to measure the angle (Ψ) from orthogonal to the plasma membrane. Compared to *C. parvum*, the greater distribution for Ψ in *T. gondii* suggests more flexibility in AV anchoring. (g, n) Relationship between AV's anchoring distance (AV_{dist}) and orientation angle Ψ. Mean ± standard deviation is reported in (c), (d), (e), (j), (k) and (l) and median for (f) and (m). Ecc: eccentricity. 3-D volumes of raw tomograms in (a, h) panels, and the subtomogram average in (b) panel are included in the repository referenced under "Data availability". Scale bars in all panels are 50 nm.

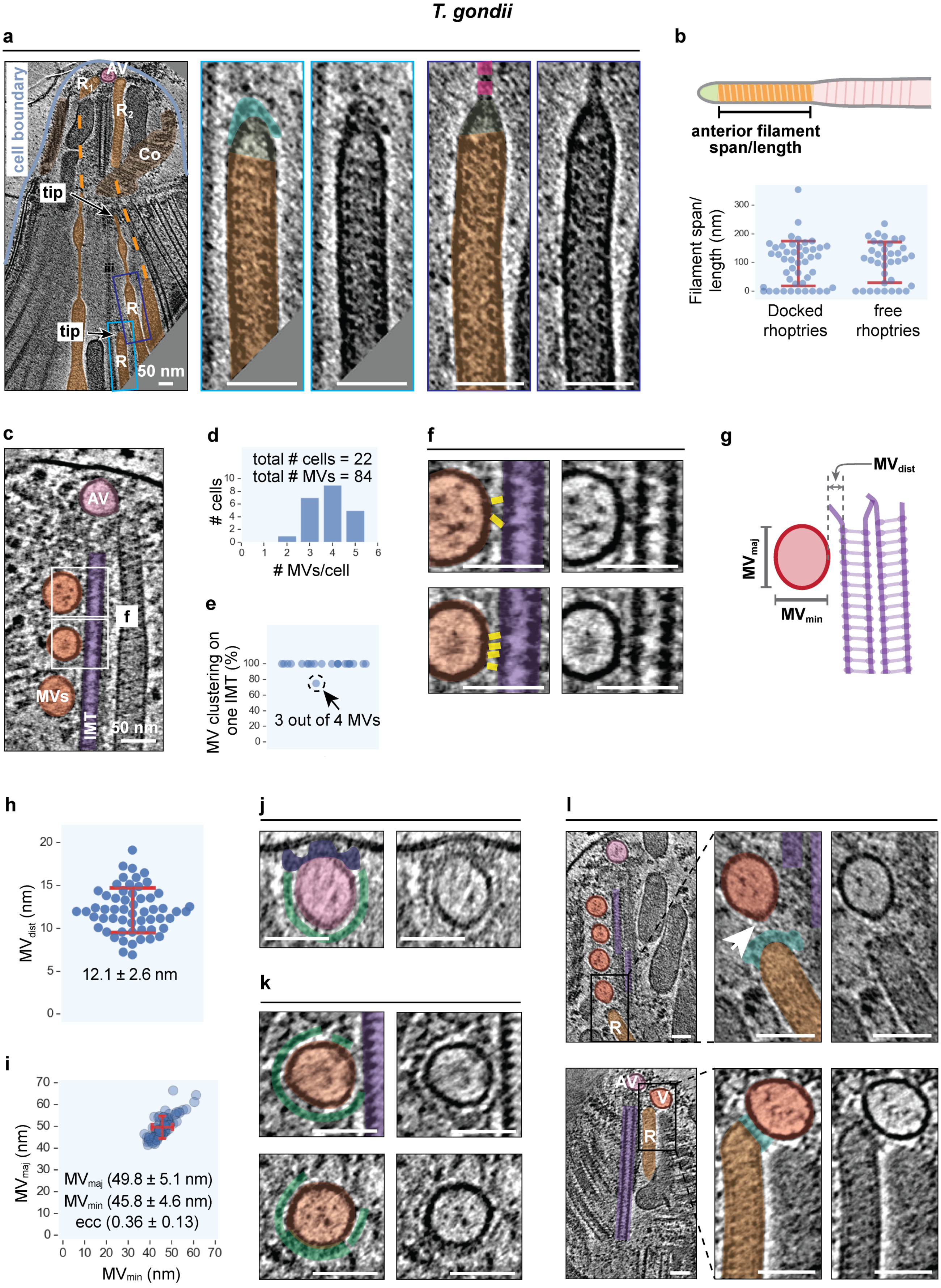

T. gondii
a
cell boundary
AV
R1
R2
Co
tip
tip
iii
R
R
50 nm
b
anterior filament span/length
Filament span/ length (nm)
300
200
100
0
Docked rhoptries
free rhoptries
c
AV
f
MVs
IMT
50 nm
d
total # cells = 22
total # MVs = 84
cells
MVs/cell
e
MV clustering on one IMT (%)
3 out of 4 MVs
f
g
MVdist
MVmaj
MVmin
h
MVdist (nm)
12.1 ± 2.6 nm
i
MVmaj (nm)
MVmin (nm)
MVmaj (49.8 ± 5.1 nm)
MVmin (45.8 ± 4.6 nm)
ecc (0.36 ± 0.13)
j
k
l
R
AV
V
R

Supplementary Fig. 7: Microtubule-associated vesicles (MVs) are possibly the precursors of the apical vesicle (AV) in *T. gondii*.

(a, b) Similarities between free (R) and docked (R_1 and R_2) rhoptries. (a) Free rhoptries show anterior filament-lined region (orange), tip (green), and tip densities (cyan). Pink dashed line indicates a rhoptry constriction. Tips of free rhoptries are marked with arrows in the larger field of view on the left. Co: conoid; AV: apical vesicle. (b) Anterior filament-lined region in free rhoptries has a similar length span as in docked rhoptries. Measurements were made on 82 rhoptries from 25 cells. (c) Several microtubule-associated vesicles (MVs; red) are seen lining up close to a pair of intraconoidal microtubules (IMTs; purple). (d) Distribution of number of MVs per cell. (e) Arrangement of MVs with respect to individual IMTs in a pair. Predominantly, all MVs in each cell are closer to the same IMT of the IMT pair. Out of 84 MVs in 22 cells, only 1 MV (out of a total 4 MVs in that particular cell) is closer to the other IMT. (f) Linker densities (yellow) between the MVs and the IMTs (enlarged views from the boxed regions in c). (g-i) MV dimensions and distances from the IMTs. MV_{dist}: shortest distance between MV membrane and the closest IMT's surface; MV_{maj}: length of the major axis of MV; MV_{min}: length of the minor axis of MV; Ecc: eccentricity. Sample size: 23 cells. (j) AVs and (k) MVs show similar proteinaceous coats (dark green). (l) MV (red; upper panels) and another similar vesicle near the cell apical tip (V; lower panels) are seen compatible for rhoptry interaction via the tip densities (cyan). Arrowhead shows deformation of the MV membrane indicative of rhoptry interaction. Source data for this figure are provided as a Source Data file. Scale bars in all panels are 50 nm.

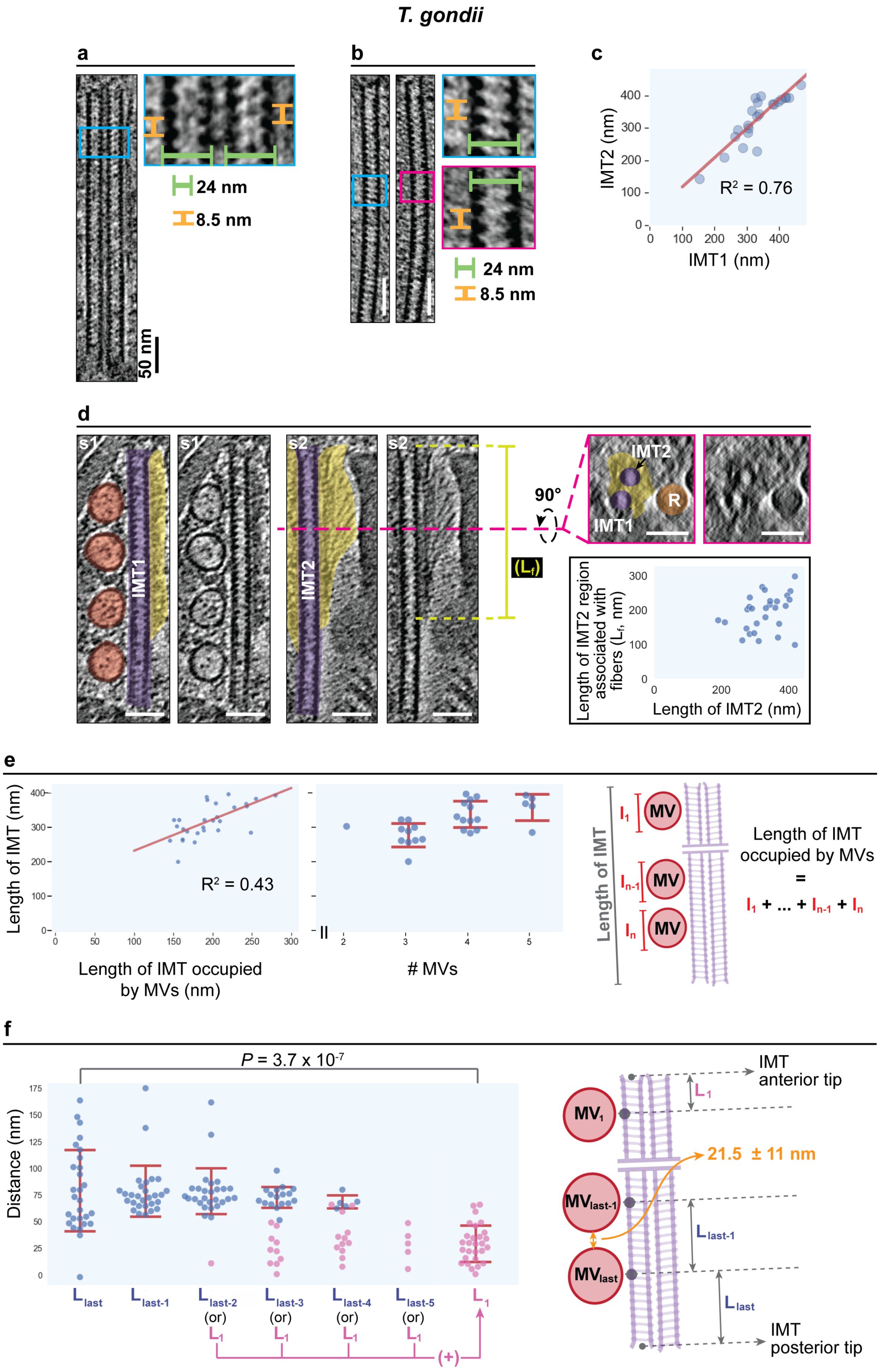
T. gondii
a
24 nm
8.5 nm
50 nm
b
24 nm
8.5 nm
c
IMT2 (nm)
IMT1 (nm)
R² = 0.76
d
s1
s2
IMT1
IMT2
90°
(Lf)
R
Length of IMT2 region associated with fibers (Lf, nm)
Length of IMT2 (nm)
e
Length of IMT (nm)
R² = 0.43
Length of IMT occupied by MVs (nm)
MVs
Length of IMT
MV
l1
ln-1
ln
Length of IMT occupied by MVs
=
l1 + … + ln-1 + ln
f
P = 3.7 x 10-7
Distance (nm)
Llast
Llast-1
Llast-2
Llast-3
Llast-4
Llast-5
(or)
L1
(+)
IMT anterior tip
MV1
21.5 ± 11 nm
MVlast-1
MVlast
IMT posterior tip

Supplementary Fig. 8: Organization of intraconoidal microtubules (IMTs) and associated structures in *T. gondii*.

(a) The intraconoidal microtubules (IMTs) are 24 nm in diameter and show a spacing of ~8.5 nm between successive subunits along their length, similar to the (b) subpellicular microtubules seen in our tomograms. Magnified images of the boxed regions on the left are shown on the right in both (a) and (b). (c) IMT measurements show a strong correlation of length between the pair of IMTs in each individual cell, while the length is highly variable from one cell to another (from ~150 nm to >400 nm). (d) Microtubule-associated vesicles (MVs; red) are arranged on one of the IMTs (IMT1; both IMTs in purple) while the other IMT (IMT2) closely interacts with a cloud of fibrous material (yellow) that forms a putative partially-assembled microtubule (in addition to the two whole IMTs). The two panels on the left (s1) and the two panels in the middle (s2) are different side view sections of the same tomogram. The two panels on the right are a top view (a section through the orthogonal plane indicated by the pink dashed line), which shows an adjacent rhoptry as well (R, orange). Inset: A plot showing that the length of IMT region associated with the fibrous material (on IMT2; L_f) is highly variable without strong correlations with IMT2's length. (e) Relationship between the length of IMT1 and its MV occupancy (left: length of IMT1 plotted versus the length cumulatively spanned by MVs; and right: length of IMT1 plotted versus the number of MVs). (f) Arrangement of MVs on IMT1. MVs are numbered from MV_1 to MV_{last} on the IMT (anterior to posterior) and their positions on the IMT are marked. Distances of these positions are measured from the IMT tips – from the anterior tip for the first MV_1 (L_1) and from the posterior tip for MV_{last} (L_{last}) – or from neighboring MV's positions (L values in between). For each cell, these distances are plotted into their corresponding column working backwards from the posterior tip. Depending on the total number of MVs in a particular cell, its L_1 value can fall into any column (other than the first), resulting in the distribution of L_1 values overall (shown in pink). The last column collects and plots these L_1 values together. Red bars show mean ± std for each distribution. The distance between the bounding membranes of neighboring MVs is also shown as mean ± std in orange. Sample size for quantifications: (e, f) n = 28 cells. Scale bars in all panels are 50 nm.

T. gondii

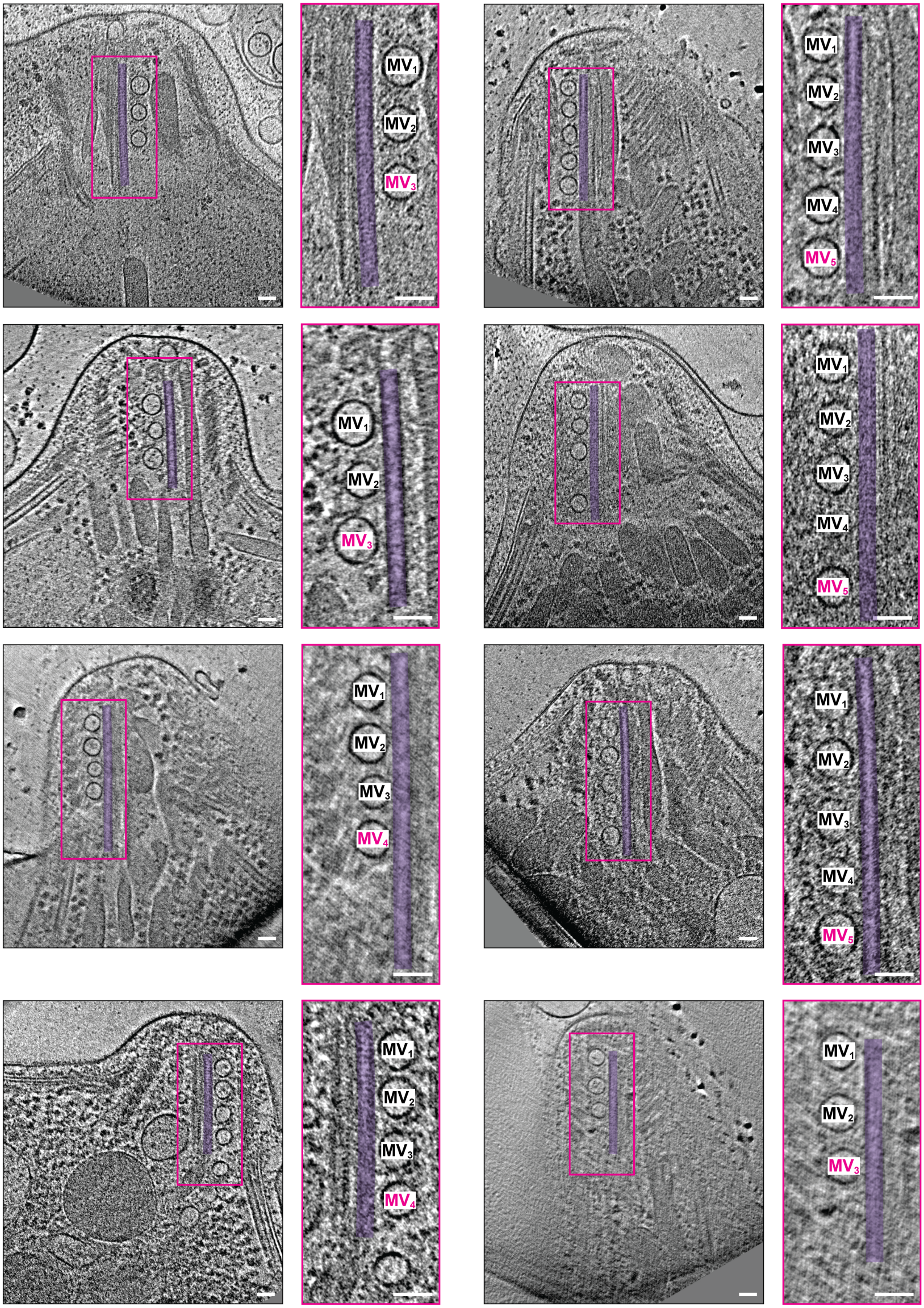
MV1
MV2
MV3
MV1
MV2
MV3
MV4
MV5
MV1
MV2
MV3
MV1
MV2
MV3
MV4
MV5
MV1
MV2
MV3
MV4
MV1
MV2
MV3
MV4
MV5
MV1
MV2
MV3
MV4
MV1
MV2
MV3

Supplementary Fig. 9: Examples for positioning of microtubule-associated vesicles (MVs) on intraconoidal microtubules (IMTs).

These eight representative cells, chosen from 28 tomograms analyzed in Supplementary Fig. 8e, f show arrangement of microtubule-associated vesicles (MVs) on *T. gondii* intraconoidal microtubules (IMTs). For each example, enlarged side views of these features (smaller panels on the right) come from the boxed region (in pink) in the corresponding cell (larger panels on the left). Purple overlay highlights the IMT that closely associates with MVs. MVs are numbered from the anterior tip of the IMT and the last MV is labeled in pink. All scale bars are 50 nm.

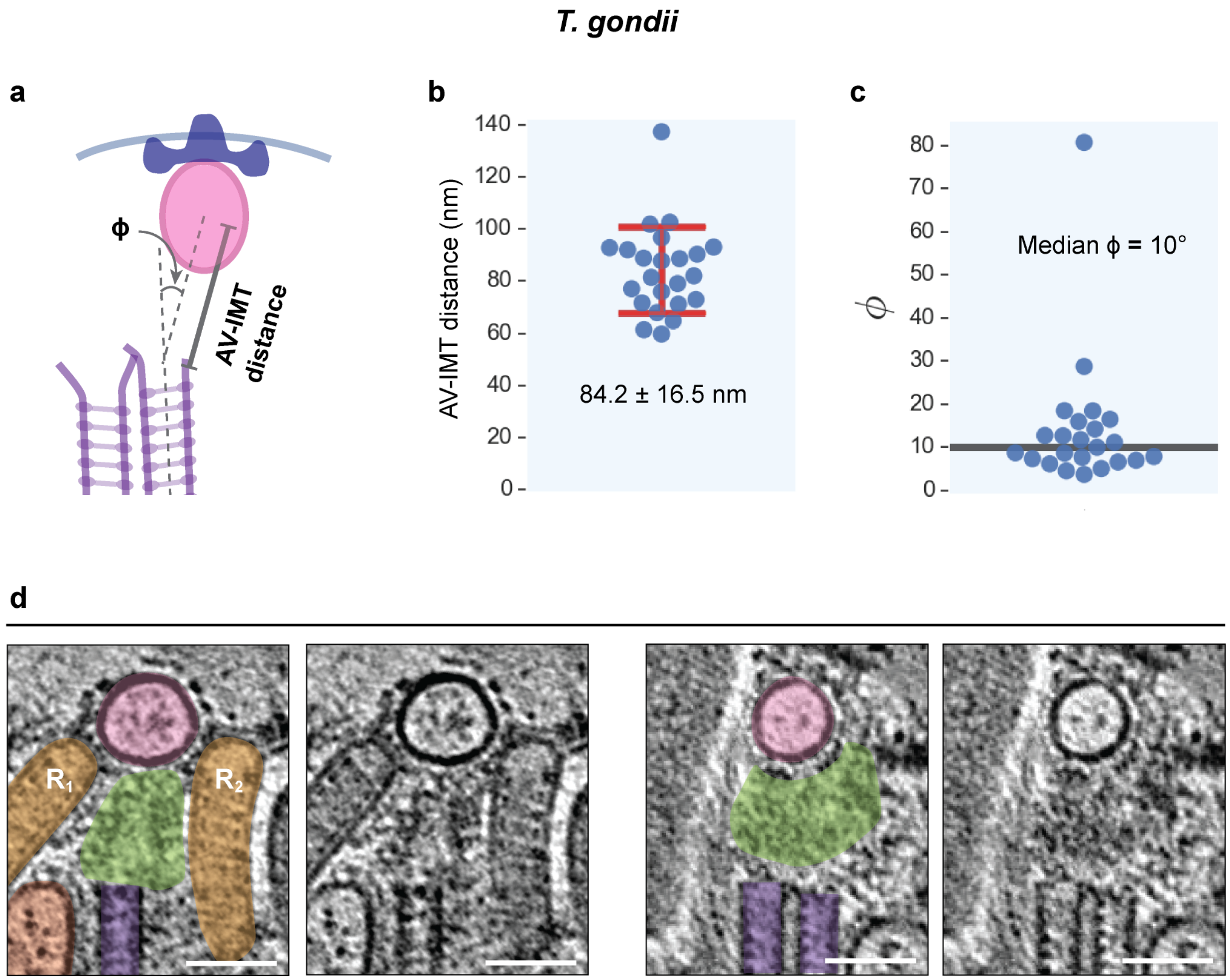

Supplementary Fig. 10: Apical vesicle (AV) is consistently organized in front of the intraconoidal microtubules (IMTs).

(a) Schematic for the measurements of the distance from the apical vesicle (AV; pink) center to the closest intraconoidal microtubule (IMT; purple) tip and its relative angle (ϕ) to the trajectory of the IMT pair. Rhoptry secretory apparatus (RSA) is represented in dark blue and the parasite plasma membrane in light blue. (b, c) Distribution of the AV-IMT distances and the angle ϕ; n = 23 cells. Red bars in (b) represents mean ± std while the black line in (c) represents the median. (d) A cloud of amorphous material (light green) sandwiched between the AV (pink) and IMT (purple) tip in two different cells along with two adjacent rhoptries (R_1, R_2; orange) in the cell on the left. Additionally, an MV (red) is highlighted. Scale bars in all panels are 50 nm.

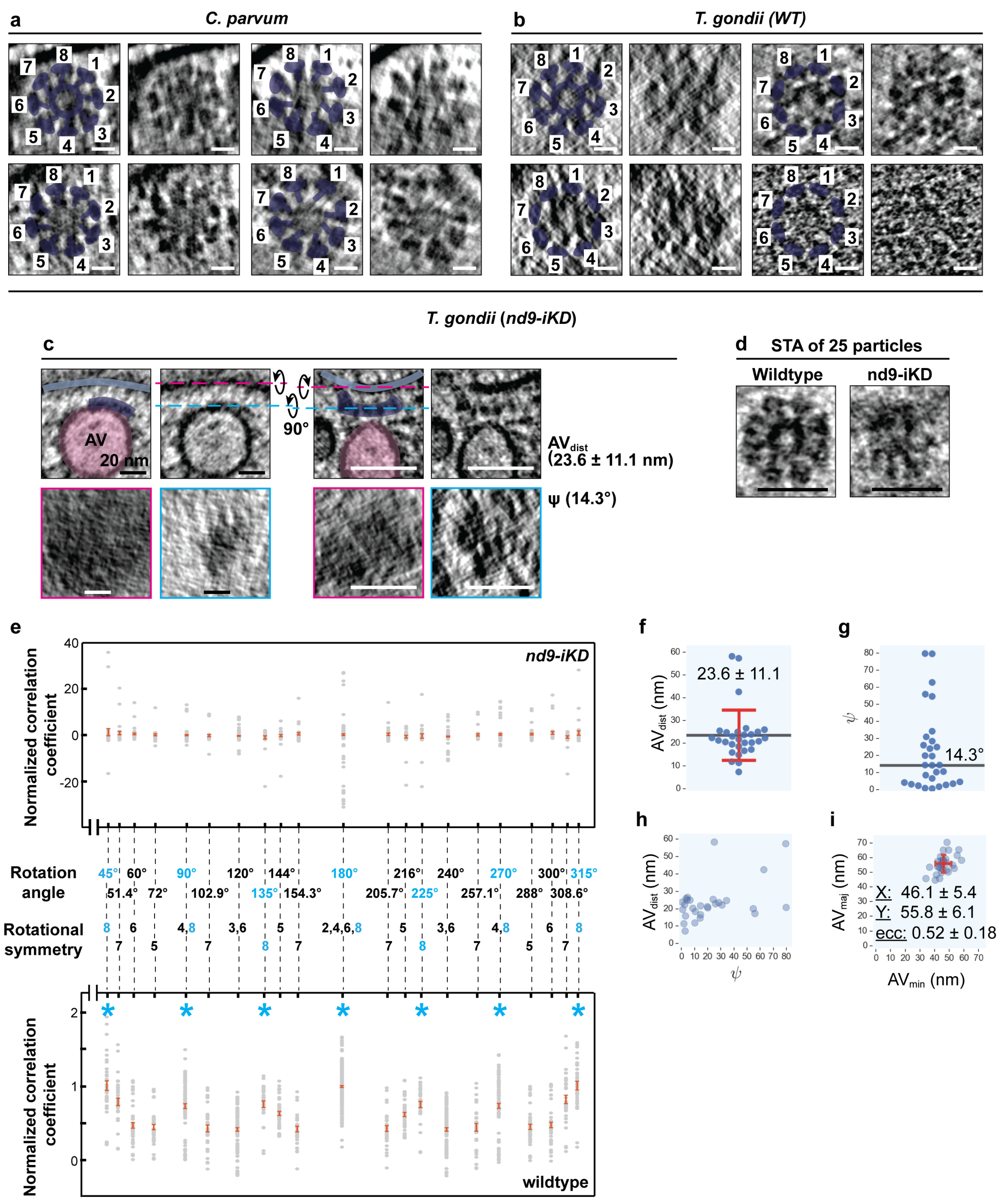

a
C. parvum
b
T. gondii (WT)
T. gondii (nd9-iKD)
c
AV
20 nm
90°
AVdist (23.6 ± 11.1 nm)
ψ (14.3°)
d
STA of 25 particles
Wildtype
nd9-iKD
e
nd9-iKD
Normalized correlation coefficient
Rotation angle
Rotational symmetry
wildtype
f
23.6 ± 11.1
AVdist (nm)
g
14.3°
h
i
X: 46.1 ± 5.4
Y: 55.8 ± 6.1
ecc: 0.52 ± 0.18
AVmaj (nm)
AVmin (nm)

Supplementary Fig. 11: Rhoptry secretory apparatus (RSA) is important for proper anchoring of apical vesicle (AV) to plasma membrane to ensure rhoptry secretion.

(a, b) More examples of top views of the rhoptry secretory apparatus (RSA; dark blue) from *C. parvum* (a) and *T. gondii* (b) showing an 8-fold rotational symmetry. (c) Improper anchoring of the apical vesicle (AV; pink) to the plasma membrane (light blue) via an inconsistent and improperly built RSA (dark blue) in Nd9 knockdown (*nd9-iKD*). Less membrane contacts (upper panels; side views) and lack of an 8-fold symmetry (lower panels; top views) can be observed. Top views are obtained from the orthogonal sectioning planes labeled in the side views with dashed lines. (d) Subtomogram average (STA; non-symmetrized) of RSAs from *nd9-iKD* cells showing a lack of 8-fold symmetry compared to that of wildtype. Top views through a representative section are shown for each. 3-D volumes of both subtomogram averages are included in the repository referenced under "Data availability". (e) Harmonic analysis for rotational symmetry in RSA. Top views of individual RSA particles were cross-correlated with themselves after rotation through various angles, each of which corresponded to particular symmetries. RSAs from *nd9-iKD* cells showed no discernable peaks at any particular angles (top), suggesting the absence of any rotational symmetry while the wildtype RSA showed local peaks at angles corresponding to 2-, 4-, and 8-fold symmetries (bottom), indicating an 8-fold symmetry. (f, g) Anchoring parameters for AV in *nd9-iKD* cells. The anchoring distance (AV_{dist}; 23.6 ± 11.1 nm) and orientation (Ψ; 14.3°) are two to three times larger and more variable compare to those of the wildtype (14 ± 2.6 nm and 4.3°; Supplementary Fig. 6l, m), indicating defects. p values in comparison to wildtype are obtained using 2-sample Kolmogorov-Smirnov tests; 4.37e-07 for AV_{dist} and 0.004 for Ψ. Moreover, in 3 of the 32 mutant cells analyzed there was no AV found near the plasma membrane (at a distance <60 nm). (h) Relationship between orientation of the AV (Ψ) and its anchoring distance in *nd9-iKD* cells suggests the importance of proximity to plasma membrane for proper orientation. (i) AV dimensions and eccentricity (ecc) in *nd9-iKD* are similar to those of wildtype cells (Supplementary Fig. 6k). Mean ± std are shown in (c), (f) and (i) while the median is shown in (g). Sample size: (f-i) n = 32 cells. Scale bars are as follows: 20 nm for example 1 on the left in (c); 50 nm for example 2 on the right in (c); and 50 nm in (d).

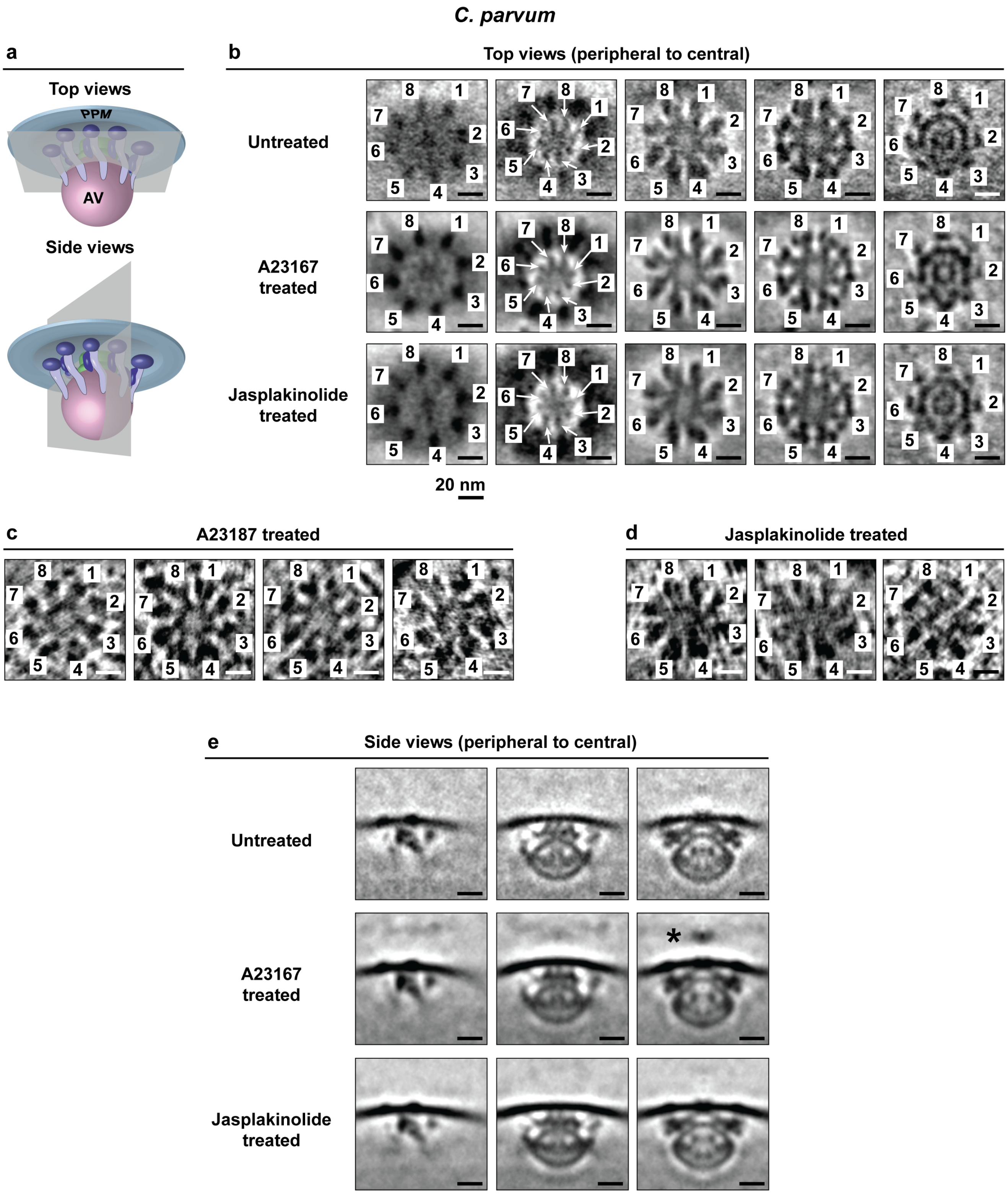
C. parvum
a
Top views
PPM
AV
Side views
b
Top views (peripheral to central)
Untreated
A23167 treated
Jasplakinolide treated
20 nm
c
A23187 treated
d
Jasplakinolide treated
e
Side views (peripheral to central)
Untreated
A23167 treated
Jasplakinolide treated
*

f

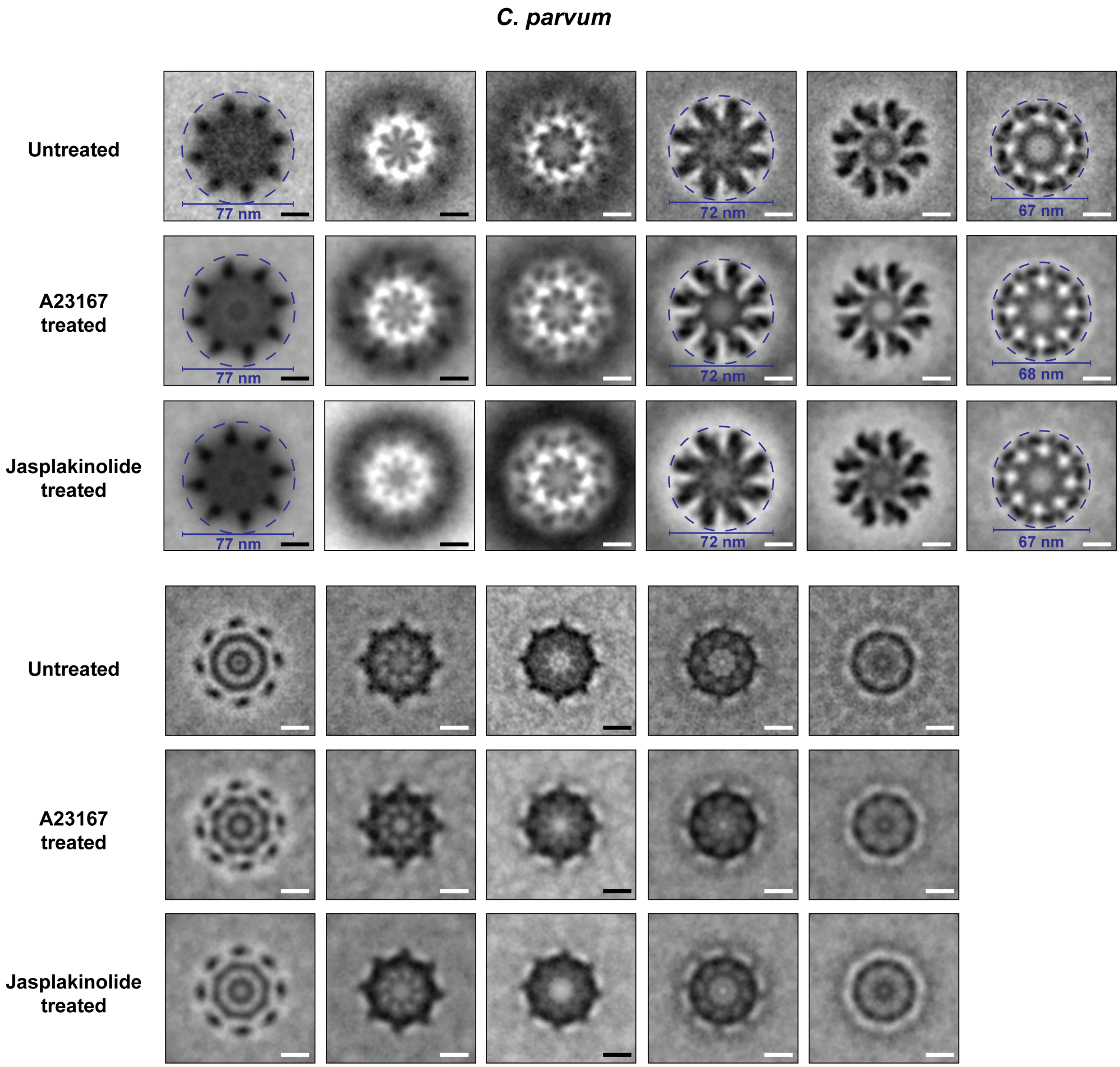
C. parvum
Untreated
A23167 treated
Jasplakinolide treated
77 nm
72 nm
67 nm
68 nm
Untreated
A23167 treated
Jasplakinolide treated

g

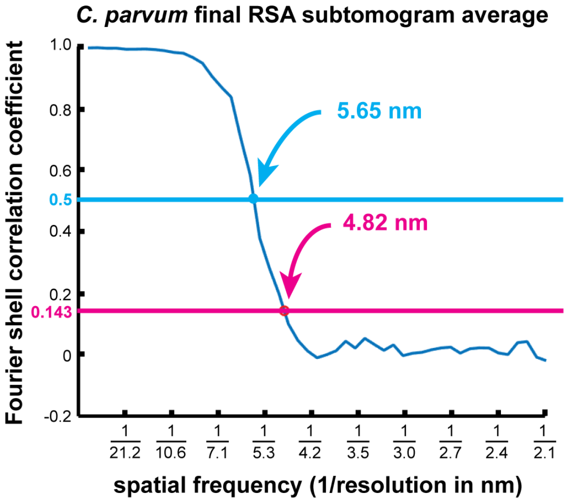
C. parvum final RSA subtomogram average
Fourier shell correlation coefficient
spatial frequency (1/resolution in nm)
5.65 nm
4.82 nm
0.5
0.143
1.0
0.8
0.6
0.4
0.2
0
-0.2
1/21.2
1/10.6
1/7.1
1/5.3
1/4.2
1/3.5
1/3.0
1/2.7
1/2.4
1/2.1

h

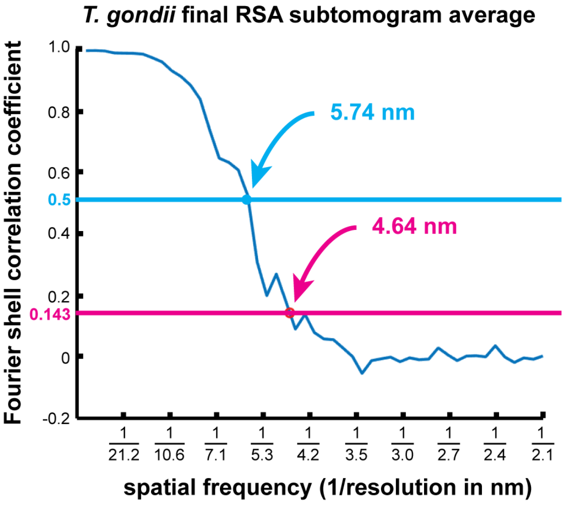
T. gondii final RSA subtomogram average
Fourier shell correlation coefficient
spatial frequency (1/resolution in nm)
5.74 nm
4.64 nm
0.5
0.143
1.0
0.8
0.6
0.4
0.2
0
-0.2
1/21.2
1/10.6
1/7.1
1/5.3
1/4.2
1/3.5
1/3.0
1/2.7
1/2.4
1/2.1

Supplementary Fig. 12: Subtomogram averages of the rhoptry secretory apparatus (RSA) in *C. parvum* in differently treated conditions.

(a) Orientations of the computational sectioning planes (gray and transparent) that reveal top views and side views of the rhoptry secretory apparatus (RSA; dark blue and purple). PPM: parasite plasma membrane (light blue) and AV: apical vesicle (pink). (b) Non-symmetrized initial subtomogram averages of RSAs from untreated, A23187-treated, or Jasplakinolide-treated cells show an overall 8-fold rotational symmetry about their central vertical axes. (c, d) Top views of individual RSAs from A23187-treated and Jasplakinolide-treated cells. The individual particles consistently show 8-fold rotational symmetry. (e, f) Subtomogram averages of untreated and drug-treated RSAs after utilizing their 8-fold symmetry (e, side views; f, top views). Asterisk (*) marked in A23167-treated sample in (e) shows an additional extracellular density whose implication is unclear. In (f), the dark blue colored dashed circle denotes the diameter of the apical rosette, which includes the central density and the eight anchor-I densities. (g) Fourier shell correlation plot for the final *C. parvum* RSA average combining all available subtomograms from all untreated and drug-treated samples. Apart from showing tomogram sections of these intermediate subtomogram averaging results, we have included their 3-D volumes in the repository referenced under “Data availability”. In total, 129 unique RSA particles were used for *C. parvum* (yielding 1032 subtomograms with its 8-fold symmetry) and 66 for *T. gondii* (yielding 528 subtomograms). The 3-D volumes of the final averages are also part of the repository referenced under “Data availability”. (h) Fourier shell correlation plot for the *T. gondii* RSA average. Scale bars in all panels are 20 nm.

C. parvum

a

Wild-type RSA average without symmetry (Supplementary Fig. 12b) → **Wild-type RSA average using 8-fold* symmetry** (Supplementary Fig. 12e, f) →

A23187-treated RSA average without symmetry (Supplementary Fig. 12b) → **A23187-treated RSA average using 8-fold* symmetry** (Supplementary Fig. 12e, f) →

Jasplakinolide-treated RSA average without symmetry (Supplementary Fig. 12b) → **Jasplakinolide-treated RSA average using 8-fold* symmetry** (Supplementary Fig. 12e, f) →

Overall final average (Fig. 3, Supplementary Movies 4 and 5)

*8-fold rotational symmetry was applied during particle cropping. Each RSA molecule yielded 8 particles by iteratively rotating it by 45 degrees.

C. parvum and T. gondii

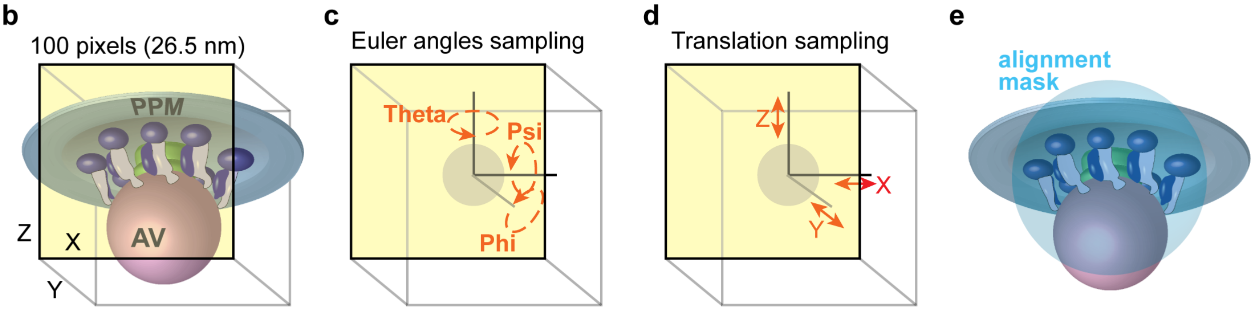

f

manually pre-oriented particles cropped → **average without alignment** (initial template) → **2-to-4 iterations of alignments using refined angular & translation sampling** → **final average after alignments**

g

C. parvum

Before 8-fold symmetry

Run #		Angular Search Range						Search	Hi Freq		Ref
		Phi		Theta		Psi		Distance	Filter		Threshold
		Max	Step	Max	Step	Max	Step		Cutoff	Sigma	
1	=>	4.5	1.5	22.5	7.5	4.5	1.5	8	0.25	0.05	80.0
2	=>	2.25	0.75	11.25	3.75	2.25	0.75	6	0.35	0.05	80.0
3	=>	1.125	0.375	5.625	1.875	1.125	0.375	4	0.4	0.05	80.0
4	=>	0.5625	0.1875	2.8125	0.9375	0.5625	0.1875	3	0.45	0.05	80.0

After 8-fold symmetry

Run #		Angular Search Range						Search	Hi Freq		Ref
		Phi		Theta		Psi		Distance	Filter		Threshold
		Max	Step	Max	Step	Max	Step		Cutoff	Sigma	
1	=>	1.125	0.375	2.8125	0.9375	1.125	0.375	4	0.4	0.05	600.0
2	=>	0.5625	0.1875	1.125	0.375	0.5625	0.1875	3	0.45	0.05	600.0

h

T. gondii

Before 8-fold symmetry

Run #		Angular Search Range						Search	Hi Freq		Ref
		Phi		Theta		Psi		Distance	Filter		Threshold
		Max	Step	Max	Step	Max	Step		Cutoff	Sigma	
1	=>	6.0	2.0	6.0	2.0	6.0	2.0	3	0.25	0.05	45.0
2	=>	3.0	1.0	3.0	1.0	3.0	1.0	2	0.3	0.05	45.0
3	=>	1.5	0.5	1.5	0.5	1.5	0.5	2	0.35	0.05	45.0

After 8-fold symmetry

Run #		Angular Search Range						Search	Hi Freq		Ref
		Phi		Theta		Psi		Distance	Filter		Threshold
		Max	Step	Max	Step	Max	Step		Cutoff	Sigma	
1	=>	3.0	1.0	3.0	1.0	3.0	1.0	4	0.25	0.05	400.0
2	=>	1.5	0.5	1.5	0.5	1.5	0.5	3	0.3	0.05	400.0
3	=>	0.75	0.25	0.75	0.25	0.75	0.25	3	0.35	0.05	400.0
4	=>	0.375	0.125	0.375	0.125	0.375	0.125	2	0.4	0.05	400.0

Supplementary Fig. 13: Subtomogram averaging schemes for the rhoptry secretory apparatus (RSA) of *C. parvum* and *T. gondii.*

(a) Scheme for generating the final subtomogram average of the rhoptry secretory apparatus (RSA; dark blue and purple) in *C. parvum* with references to results of intermediate steps (in gray). (b-h) Details of subtomogram averaging procedure for each step in (a) including (b) subtomogram extraction, (c, d) angular and translational search during alignment, (e) spherical alignment mask, which encompasses the parasite plasma membrane (PPM; light blue), RSA and part of the apical vesicle (AV; pink), and shown in cyan, (f) alignment procedure using PEET, and (g, h) search parameters for alignment of *C. parvum* and *T. gondii* RSAs. (f) Subtomograms were manually picked in a pre-oriented fashion and averaged without alignments to generate a template for subsequent alignments. Several iterations of refined search (for three Euler angles and translations) were performed to arrive at the final subtomogram orientations and average for each step in (a) including the overall final averages for *C. parvum* and *T. gondii* RSAs (shown in Fig. 3). For these final averages, as with the individual sub-groups of *C. parvum* RSA (untreated and 2 different drug treatments), we first aligned all subtomograms without utilizing the 8-fold symmetry (search parameters on the left in panels (g) and (h)) following which we expanded the number of subtomograms 8-fold and realigned (search parameters on the right in panels (g) and (h)). 3-D volumes of all the intermediate and final averages are in the repository referenced under “Data availability” for Supplementary Fig. 12.

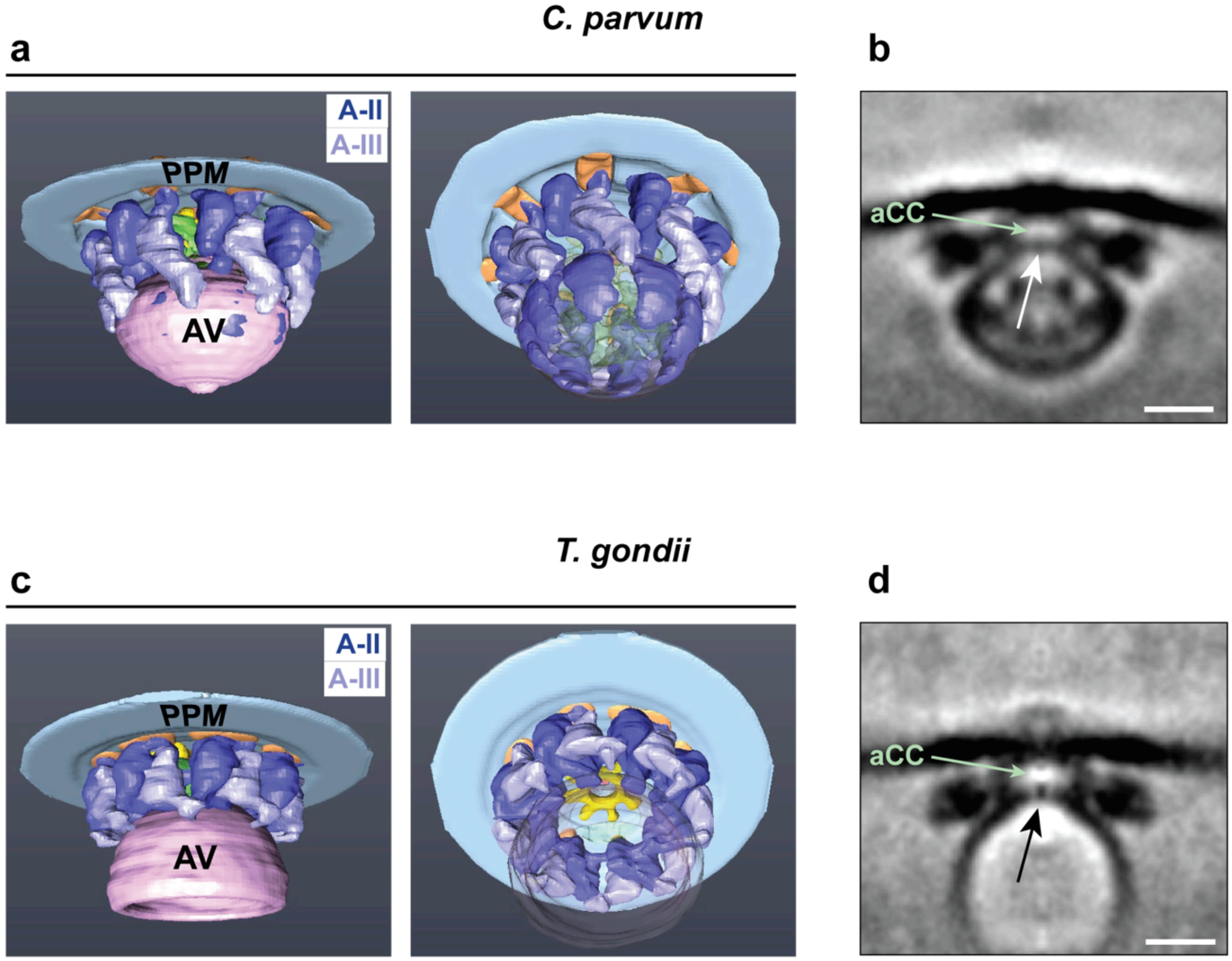

Supplementary Fig. 14: Ultrastructures of rhoptry secretory apparatus (RSA) in *C. parvum* and *T. gondii* – additional interpretations.

(a, c) Anchor-II (A-II; dark blue) and anchor-III (A-III; purple) of the rhoptry secretory apparatus (RSA) interact with the apical vesicle (AV; pink), but the arms of A-II and A-III are shorter in *T. gondii* (c) compared to those in *C. parvum* (a). A-II in *C. parvum* (a) interacts more extensively with the AV membrane compared to that in *T. gondii* (c), likely contributing to the distinct shape of *C. parvum* AV (a, left panel). PPM: parasite plasma membrane (light blue). (b, d) Side view (central section) of AV and RSA in *C. parvum* (b) and *T. gondii* (d) showing protein densities (white and black arrows, respectively) embedded in the AV membrane at the entrance of the anterior central channel (aCC; green arrow). Scale bars in all panels are 20 nm.

T. gondii

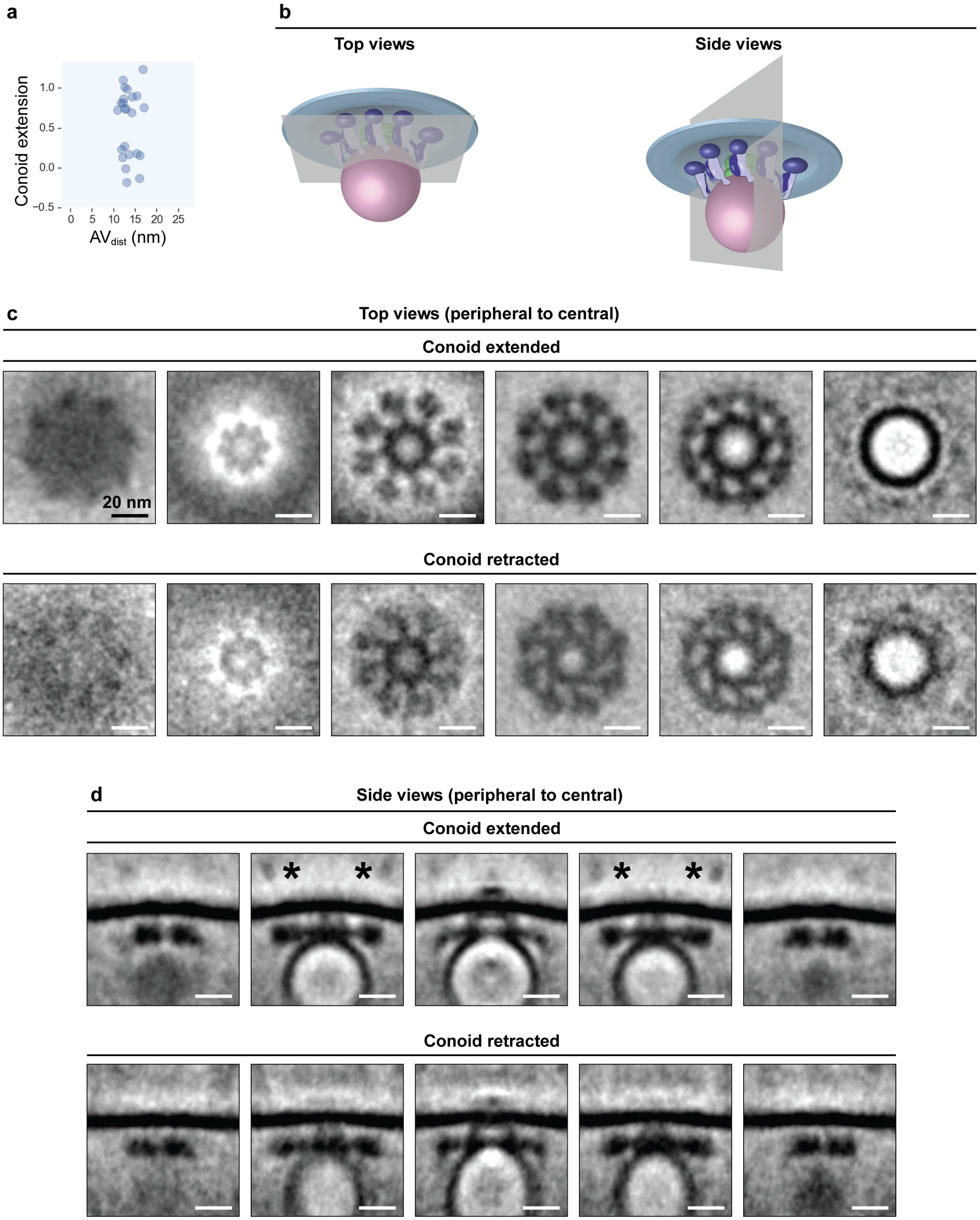

a
Conoid extension
1.0
0.5
0.0
−0.5
0
5
10
15
20
25
AV_dist (nm)
b
Top views
Side views
c
Top views (peripheral to central)
Conoid extended
20 nm
Conoid retracted
d
Side views (peripheral to central)
Conoid extended
*
*
*
*
Conoid retracted

Supplementary Fig. 15: Relationship between conoid extension and the ultrastructure of rhoptry secretory apparatus (RSA).

(a) Scatterplot exploring the relationship between conoid extension and anchoring distance of the apical vesicle (AV) from the parasite plasma membrane (PPM) in individual cells (25 cells in total). The two populations – one with retracted conoid (9 cells) and the other with extended conoid (16 cells) – are evident from the scatterplot. However, anchoring of AV is shown to be independent of conoid extension. (b) Schematic showing the orientation of sectioning planes (gray and transparent) to obtain the top views in (c) and the side views in (d). PPM is shown in light blue, AV in pink, and the rhoptry secretory apparatus (RSA) in dark blue and purple. (c, d) Subtomogram averages of RSA in cells with the conoid either extended or retracted. No large-scale conformational differences are seen between these two ultrastructures. Asterisks (*) in (d) indicate the appearance of some extracellular densities in conoid extended sample whose significance is unknown. Scale bars in all panels are 20 nm.

3. Supplementary Tables

	Mean	Std	95% confidence interval
**Rhoptry 1**	84.58	4.11	83.27 - 85.89
**Rhoptry 2**	75.77	3.20	74.85 - 76.68
**Rhoptry 3**	79.37	3.93	78.18 - 80.56
**Rhoptry 4**	83.50	9.98	80.55 - 86.45
**Rhoptry 5**	77.25	2.70	76.40 - 78.10
**Rhoptry 6**	78.00	5.78	76.16 - 79.84
**Rhoptry 7**	86.82	8.65	83.21 - 90.44
**Rhoptry 8**	87.67	8.17	84.88 - 90.45
**Rhoptry 9**	96.35	6.64	94.47 - 98.23
**Rhoptry 10**	102.61	15.53	96.64 - 108.58
**Rhoptry 11**	83.56	7.40	80.32 - 86.81
**Rhoptry 12**	72.50	29.17	64.64 - 80.35
**Rhoptry 13**	82.77	4.79	81.27 - 84.28
**Rhoptry 14**	80.70	3.18	79.79 - 81.61
**Rhoptry 15**	79.18	9.26	76.56 - 81.80
**Rhoptry 16**	79.00	8.76	75.87 - 82.13
**Rhoptry 17**	80.35	2.15	79.69 - 81.02
**Rhoptry 18**	87.45	4.47	86.17 - 88.73
**Rhoptry 19**	83.88	4.62	82.67 - 85.09
**Rhoptry 20**	82.67	5.37	80.43 - 84.91
**Rhoptry 21**	82.72	5.67	81.06 - 84.38
**Rhoptry 22**	80.72	6.18	78.67 - 82.77
**Rhoptry 23**	81.19	3.37	80.17 - 82.21
**Rhoptry 24**	82.61	4.05	81.51 - 83.71
**Rhoptry 25**	83.53	3.31	82.67 - 84.39
**Rhoptry 26**	82.39	6.11	80.24 - 84.54
**All rhoptries**	**82.58**	**10.58**	**81.94 - 83.23**

Supplementary Table 1: Measurements of diameter from individual rhoptry necks in *C. parvum.*

4. Supplementary References

1 Beisson, J., Cohen, J., Lefort-Tran, M., Pouphile, M. & Rossignol, M. Control of membrane fusion in exocytosis. Physiological studies on a Paramecium mutant blocked in the final step of the trichocyst extrusion process. *J Cell Biol* **85**, 213-227, doi:10.1083/jcb.85.2.213 (1980).

2 Beisson, J., Lefort-Tran, M., Pouphile, M., Rossignol, M. & Satir, B. Genetic analysis of membrane differentiation in Paramecium. Freeze-fracture study of the trichocyst cycle in wild-type and mutant strains. *J Cell Biol* **69**, 126-143, doi:10.1083/jcb.69.1.126 (1976).

3 Knoll, G., Braun, C. & Plattner, H. Quenched flow analysis of exocytosis in Paramecium cells: time course, changes in membrane structure, and calcium requirements revealed after rapid mixing and rapid freezing of intact cells. *J Cell Biol* **113**, 1295-1304, doi:10.1083/jcb.113.6.1295 (1991).

4 Matt, H., Bilinski, M. & Plattner, H. Adenosinetriphosphate, calcium and temperature requirements for the final steps of exocytosis in Paramecium cells. *J Cell Sci* **32**, 67-86 (1978).

5 Olbricht, K., Plattner, H. & Matt, H. Synchronous exocytosis in Paramecium cells. II. Intramembranous changes analysed by freeze-fracturing. *Exp Cell Res* **151**, 14-20, doi:10.1016/0014-4827(84)90351-3 (1984).

6 Plattner, H. Intramembraneous changes on cationophore-triggered exocytosis in Paramecium. *Nature* **252**, 722-724, doi:10.1038/252722a0 (1974).

7 Plattner, H. Membrane behaviour during exocytosis. *Cell Biol Int Rep* **5**, 435-459, doi:10.1016/0309-1651(81)90165-x (1981).

8 Plattner, H., Miller, F. & Bachmann, L. Membrane specializations in the form of regular membrane-to-membrane attachment sites in Paramecium. A correlated freeze-etching and ultrathin-sectioning analysis. *J Cell Sci* **13**, 687-719 (1973).

9 Satir, B. Membrane events during the secretory process. *Symp Soc Exp Biol*, 399-418 (1974).

10 Satir, B., Schooley, C. & Satir, P. Membrane reorganization during secretion in Tetrahymena. *Nature* **235**, 53-54, doi:10.1038/235053a0 (1972).

11 Satir, B., Schooley, C. & Satir, P. Membrane fusion in a model system. Mucocyst secretion in Tetrahymena. *J Cell Biol* **56**, 153-176, doi:10.1083/jcb.56.1.153 (1973).

12 Satir, B. H. & Oberg, S. G. Paramecium fusion rosettes: possible function as Ca2+ gates. *Science* **199**, 536-538, doi:10.1126/science.341312 (1978).

13 Aquilini, E. *et al.* An Alveolata secretory machinery adapted to parasite host cell invasion. *Nat Microbiol* **6**, 425-434, doi:10.1038/s41564-020-00854-z (2021).

14 Boothroyd, J. C. & Dubremetz, J. F. Kiss and spit: the dual roles of Toxoplasma rhoptries. *Nat Rev Microbiol* **6**, 79-88, doi:10.1038/nrmicro1800 (2008).

15 Dubois, D. J. & Soldati-Favre, D. Biogenesis and secretion of micronemes in Toxoplasma gondii. *Cell Microbiol* **21**, e13018, doi:10.1111/cmi.13018 (2019).

16 Dubremetz, J. F. Rhoptries are major players in Toxoplasma gondii invasion and host cell interaction. *Cell Microbiol* **9**, 841-848, doi:10.1111/j.1462-5822.2007.00909.x (2007).

17 Hakansson, S., Charron, A. J. & Sibley, L. D. Toxoplasma evacuoles: a two-step process of secretion and fusion forms the parasitophorous vacuole. *EMBO J* **20**, 3132-3144, doi:10.1093/emboj/20.12.3132 (2001).

18 Suss-Toby, E., Zimmerberg, J. & Ward, G. E. Toxoplasma invasion: the parasitophorous vacuole is formed from host cell plasma membrane and pinches off via a fission pore. *Proc Natl Acad Sci U S A* **93**, 8413-8418, doi:10.1073/pnas.93.16.8413 (1996).

19 Matt, H., Plattner, H., Reichel, K., Lefort-Tran, M. & Beisson, J. Genetic dissection of the final exocytosis steps in Paramecium tetraurelia cells: trigger analyses. *J Cell Sci* **46**, 41-60 (1980).

20 Plattner, H. *et al.* Genetic dissection of the final exocytosis steps in Paramecium tetraurelia cells: cytochemical determination of Ca2+-ATPase activity over performed exocytosis sites. *J Cell Sci* **46**, 17-40 (1980).

21 Coleman, B. I. *et al.* A Member of the Ferlin Calcium Sensor Family Is Essential for Toxoplasma gondii Rhoptry Secretion. *mBio* **9**, doi:10.1128/mBio.01510-18 (2018).

22 Mondragon, R. & Frixione, E. Ca(2+)-dependence of conoid extrusion in Toxoplasma gondii tachyzoites. *J Eukaryot Microbiol* **43**, 120-127, doi:10.1111/j.1550-7408.1996.tb04491.x (1996).

23 Harris, J. R., Adrian, M. & Petry, F. Structure of the Cryptosporidium parvum microneme: a metabolically and osmotically labile apicomplexan organelle. *Micron* **34**, 65-78, doi:10.1016/s0968-4328(03)00020-9 (2003).
