# Supplementary material for: In situ ultrastructures of two evolutionarily distant apicomplexan rhoptry secretion systems
Source: Nat Commun. 2021 Aug 17;12:4983. doi: 10.1038/s41467-021-25309-9 (PMC8371170; doi:10.1038/s41467-021-25309-9)
Supplement: Supplementary file 3 — Reporting Summary [file 41467_2021_25309_MOESM3_ESM.pdf]

nature research

Corresponding author(s): Yi-Wei Chang

Last updated by author(s): 14 July, 2021

Reporting Summary

Nature Research wishes to improve the reproducibility of the work that we publish. This form provides structure for consistency and transparency in reporting. For further information on Nature Research policies, see our Editorial Policies and the Editorial Policy Checklist.

Statistics

For all statistical analyses, confirm that the following items are present in the figure legend, table legend, main text, or Methods section.

n/a	Confirmed	
☐	☒	The exact sample size (*n*) for each experimental group/condition, given as a discrete number and unit of measurement
☐	☒	A statement on whether measurements were taken from distinct samples or whether the same sample was measured repeatedly
☐	☒	The statistical test(s) used AND whether they are one- or two-sided<br>*Only common tests should be described solely by name; describe more complex techniques in the Methods section.*
☒	☐	A description of all covariates tested
☒	☐	A description of any assumptions or corrections, such as tests of normality and adjustment for multiple comparisons
☐	☒	A full description of the statistical parameters including central tendency (e.g. means) or other basic estimates (e.g. regression coefficient) AND variation (e.g. standard deviation) or associated estimates of uncertainty (e.g. confidence intervals)
☐	☒	For null hypothesis testing, the test statistic (e.g. *F*, *t*, *r*) with confidence intervals, effect sizes, degrees of freedom and *P* value noted<br>*Give P values as exact values whenever suitable.*
☒	☐	For Bayesian analysis, information on the choice of priors and Markov chain Monte Carlo settings
☒	☐	For hierarchical and complex designs, identification of the appropriate level for tests and full reporting of outcomes
☐	☒	Estimates of effect sizes (e.g. Cohen's *d*, Pearson's *r*), indicating how they were calculated

Our web collection on statistics for biologists contains articles on many of the points above.

Software and code

Policy information about availability of computer code

Data collection	Tilt series acquisition - SerialEM 3.7 and 3.8.
Data analysis	Tomogram reconstruction, subtomogram averaging and modeling - IMOD (v4.11) and PEET (v1.13); Segmentation - Amira 2019; Analyses and Graphing - MATLAB R2020b and Python 3.5 (Numpy 1.19.2, Matplotlib 3.3.2, Scipy 1.6.2, Seaborn 0.11.0).

For manuscripts utilizing custom algorithms or software that are central to the research but not yet described in published literature, software must be made available to editors and reviewers. We strongly encourage code deposition in a community repository (e.g. GitHub). See the Nature Research guidelines for submitting code & software for further information.

Data

Policy information about availability of data

All manuscripts must include a data availability statement. This statement should provide the following information, where applicable:

- Accession codes, unique identifiers, or web links for publicly available datasets
- A list of figures that have associated raw data
- A description of any restrictions on data availability

Additional data generated in this study are provided in the Supplementary Information and Source Data file. Source data include tomogram sections without any color annotations. Three-dimensional tomogram volumes are also made available through an online repository – https://figshare.com/projects/In_situ_ultrastructures_of_two_evolutionarily_distant_apicomplexan_rhoptry_secretion_systems/112917; individual DOI links include https://doi.org/10.6084/m9.figshare.14527794.v3 (for Supplementary Fig. 2), https://doi.org/10.6084/m9.figshare.14538339.v5 (for Supplementary Fig. 5), https://doi.org/10.6084/m9.figshare.14534109.v2 (for Supplementary Fig. 6), https://doi.org/10.6084/m9.figshare.14527890.v2 (for Supplementary Fig. 11), and https://doi.org/10.6084/m9.figshare.14527911.v4 (for Supplementary Fig. 12).
Any remaining minor datasets and analyses generated for the current study are available from the corresponding author upon request.

Field-specific reporting

Please select the one below that is the best fit for your research. If you are not sure, read the appropriate sections before making your selection.

- [x] Life sciences
- [ ] Behavioural & social sciences
- [ ] Ecological, evolutionary & environmental sciences

For a reference copy of the document with all sections, see nature.com/documents/nr-reporting-summary-flat.pdf

Life sciences study design

All studies must disclose on these points even when the disclosure is negative.

Sample size	No statistical method was used to predetermine sample size. Sample sizes were instead arbitrary chosen based on previous, similar tomographic analyses - Science Advances 26 Mar 2021: Vol. 7, no. 13, eabc6345 (DOI: 10.1126/sciadv.abc6345)
Data exclusions	No data was excluded.
Replication	We obtained a total of 285 tomograms for C. parvum (119 for untreated, 57 for A23187-treated, and 109 for Jasplakinolide-treated) and 100 for T. gondii from multiple frozen grids that were imaged over several sessions (at least 2 multiple-day sessions for each sample). All attempts at replication were successful.
Randomization	Samples (parasites) were allocated randomly into experimental groups to either receive a drug treatment or be treated as negative control.
Blinding	No blinding efforts were taken during experiments since the experimental design involved specific drug treatments and imaging that are independent of experimenter bias. No blinding efforts were again taken during analyses since ground rules were set to eliminate bias.

Reporting for specific materials, systems and methods

We require information from authors about some types of materials, experimental systems and methods used in many studies. Here, indicate whether each material, system or method listed is relevant to your study. If you are not sure if a list item applies to your research, read the appropriate section before selecting a response.

Materials & experimental systems

n/a	Involved in the study	
x		Antibodies
	x	Eukaryotic cell lines
x		Palaeontology and archaeology
x		Animals and other organisms
x		Human research participants
x		Clinical data
x		Dual use research of concern

Methods

n/a	Involved in the study	
x		ChIP-seq
x		Flow cytometry
x		MRI-based neuroimaging

Eukaryotic cell lines

Policy information about cell lines

Cell line source(s)	Human foreskin fibroblasts (HFF) – ATCC, CRL 1634
Authentication	No additional authentication done by us
Mycoplasma contamination	Not tested for Mycoplasma
Commonly misidentified lines (See ICLAC register)	None
