# Supplementary material for: In situ ultrastructures of two evolutionarily distant apicomplexan rhoptry secretion systems
Source: Nat Commun. 2021 Aug 17;12:4983. doi: 10.1038/s41467-021-25309-9 (PMC8371170; doi:10.1038/s41467-021-25309-9)
Supplement: Supplementary file 4 — Description of Additional Supplementary Files [file 41467_2021_25309_MOESM4_ESM.pdf]

Description of Additional Supplementary Files

File Name: Supplementary Movie 1
Description: Organization of C. parvum’s apical end.

File Name: Supplementary Movie 2
Description: Organization of T. gondii’s apical end.

File Name: Supplementary Movie 3
Description: Three-dimensional visualization of T. gondii’s anterior filament geometry.

File Name: Supplementary Movie 4
Description: Three-dimensional visualization of C. parvum’s rhoptry secretory apparatus.

File Name: Supplementary Movie 5
Description: Three-dimensional visualization of T. gondii’s rhoptry secretory apparatus.
