# Supplementary material for: In situ ultrastructures of two evolutionarily distant apicomplexan rhoptry secretion systems
Source: Nat Commun. 2021 Aug 17;12:4983. doi: 10.1038/s41467-021-25309-9 (PMC8371170; doi:10.1038/s41467-021-25309-9)

Source Data: Raw tomogram sections presented in this study without color overlays.

Raw tomogram sections in Main Figures

Fig. 1b

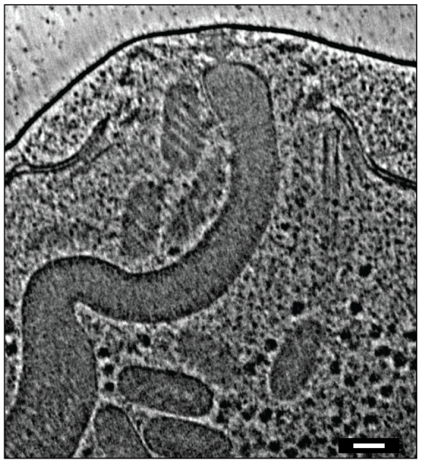

Fig. 1f

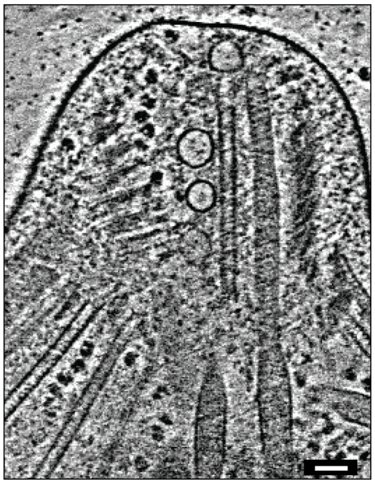

Fig. 2a

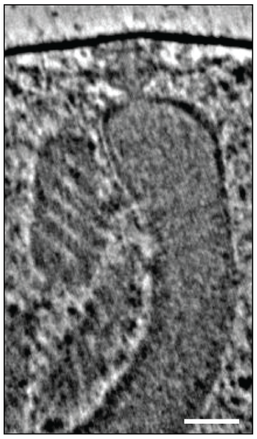

Fig. 2f

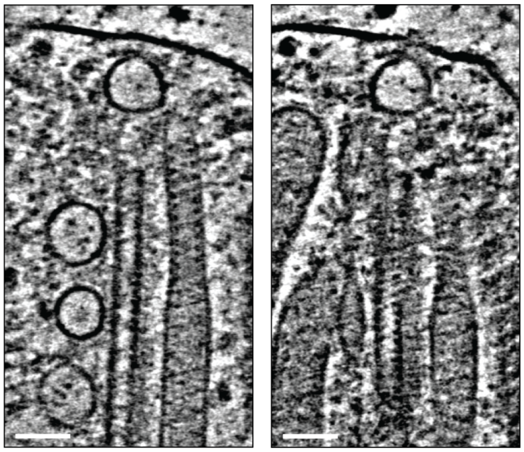

Fig. 3b

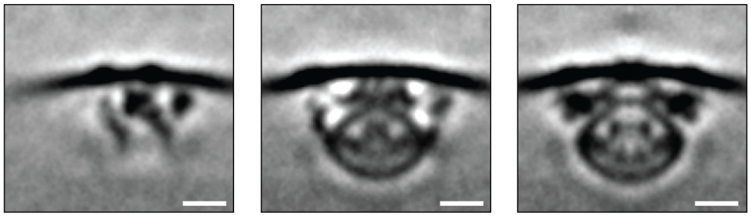

Fig. 3f

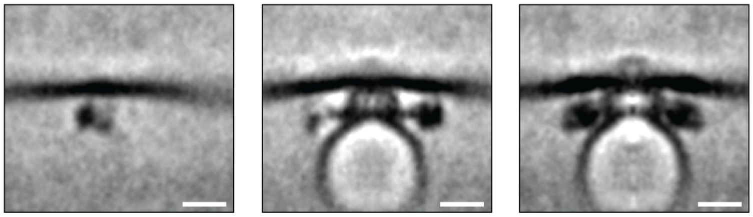

Fig. 3c

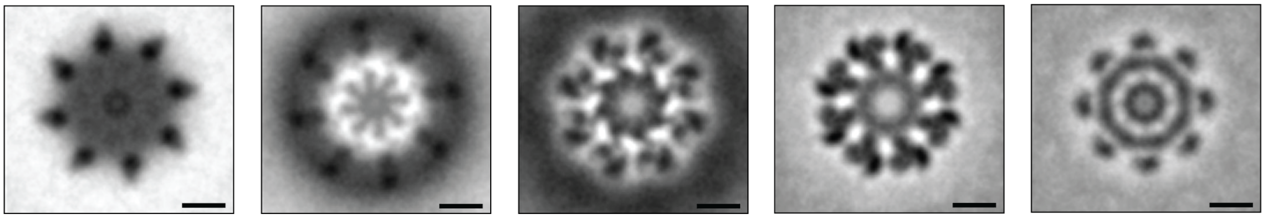

Fig. 3g

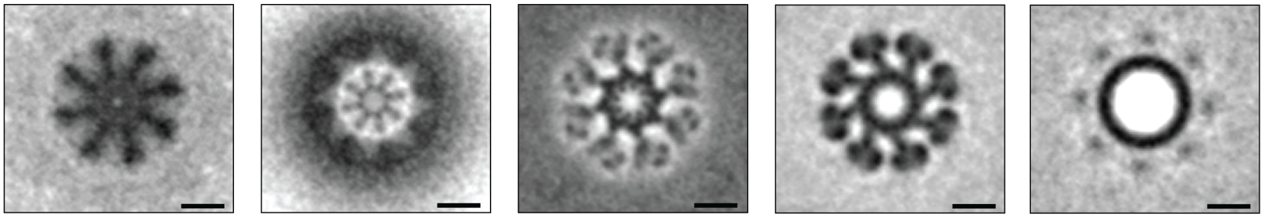

Raw tomogram sections in Supplementary Figures

Supplementary Fig. 2b

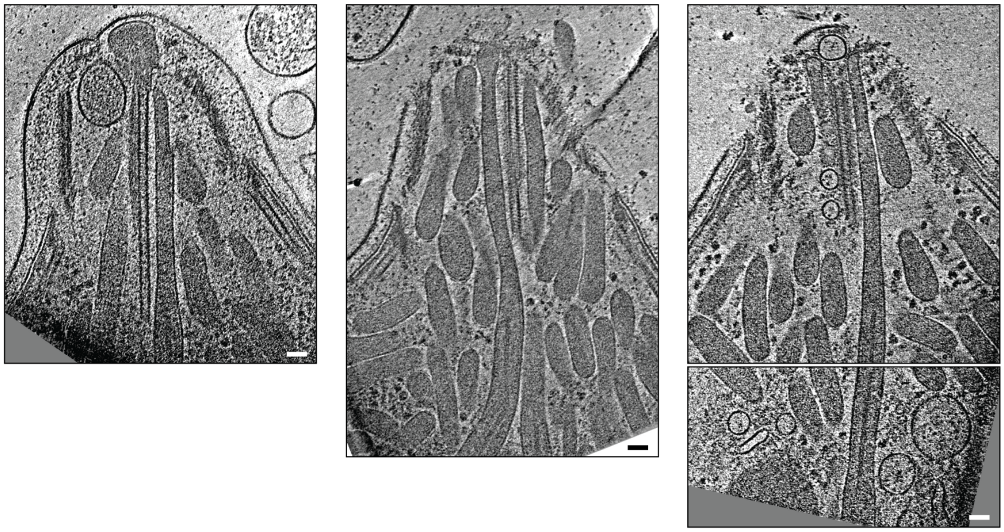

Supplementary Fig. 4

a

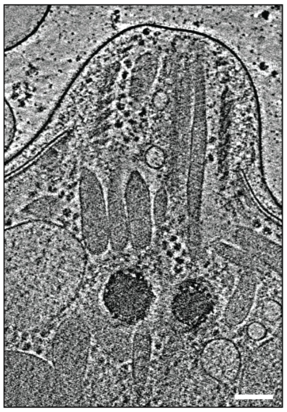

b

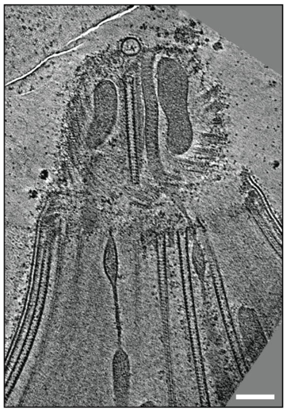

c

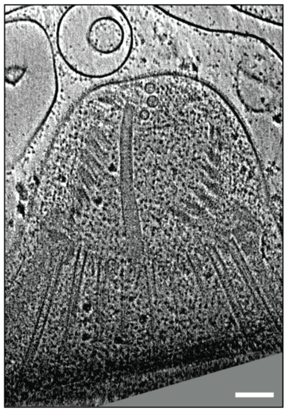

d

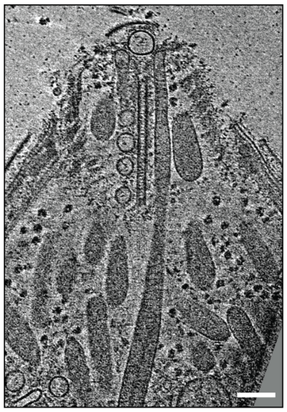

e

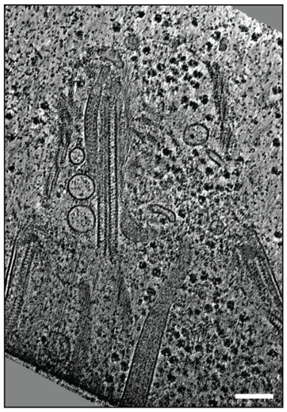

f

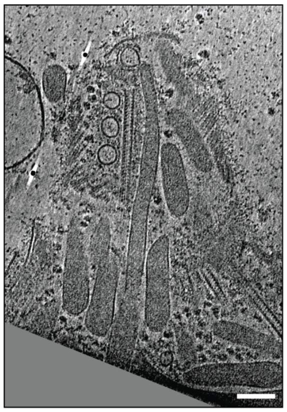

Supplementary Fig. 7a

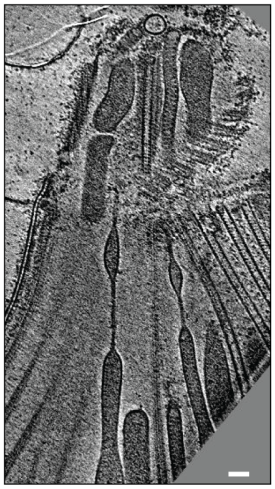

Supplementary Fig. 7l

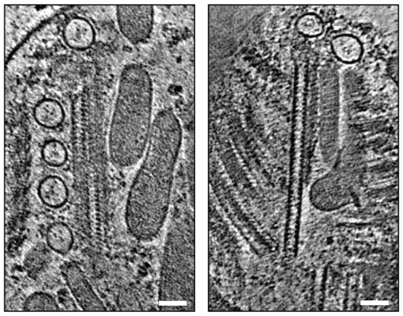

Supplement: Supplementary file 10 — Source Data [file 41467_2021_25309_MOESM10_ESM.pdf]
